# Advances in Natural Products from the Marine-Sponge-Associated Microorganisms with Antimicrobial Activity in the Last Decade

**DOI:** 10.3390/md21040236

**Published:** 2023-04-12

**Authors:** Jiaqi Liang, Jianglian She, Jun Fu, Jiamin Wang, Yuxiu Ye, Bin Yang, Yonghong Liu, Xuefeng Zhou, Huaming Tao

**Affiliations:** 1 CAS Key Laboratory of Tropical Marine Bio-Resources and Ecology, Guangdong Key Laboratory of Marine Materia Medica, South China Sea Institute of Oceanology, Chinese Academy of Sciences, Guangzhou 510301, Chinayangbin@scsio.ac.cn (B.Y.); yonghongliu@scsio.ac.cn (Y.L.); 2 University of Chinese Academy of Sciences, Beijing 100049, China; 3 Guangdong Provincial Key Laboratory of Chinese Medicine Pharmaceutics, School of Traditional Chinese Medicine, Southern Medical University, Guangzhou 510515, China

**Keywords:** sponge, associated microorganisms, secondary metabolites, antimicrobial activity

## Abstract

Microorganisms are the dominating source of food and nutrition for sponges and play an important role in sponge structure, chemical defense, excretion and evolution. In recent years, plentiful secondary metabolites with novel structures and specific activities have been identified from sponge-associated microorganisms. Additionally, as the phenomenon of the drug resistance of pathogenic bacteria is becoming more and more common, it is urgent to discover new antimicrobial agents. In this paper, we reviewed 270 secondary metabolites with potential antimicrobial activity against a variety of pathogenic strains reported in the literature from 2012 to 2022. Among them, 68.5% were derived from fungi, 23.3% originated from actinomycetes, 3.7% were obtained from other bacteria and 4.4% were discovered using the co-culture method. The structures of these compounds include terpenoids (13%), polyketides (51.9%), alkaloids (17.4%), peptides (11.5%), glucosides (3.3%), etc. Significantly, there are 124 new compounds and 146 known compounds, 55 of which have antifungal activity in addition to antipathogenic bacteria. This review will provide a theoretical basis for the further development of antimicrobial drugs.

## 1. Introduction

Marine sponges, the most primitive multicellular metazoan animals, are sessile organisms that efficiently filter feed organisms from the surrounding water [1]. They live in a high-salinity, high-pressure, light-avoiding, anoxic and oligotrophic environment. As natural microbial fermenters, sponges harbor a large community of diverse microorganisms that represent up to 50–60% of the sponge biomass [2]. In addition, these microorganisms are rich in silent genes due to the special living environment, which can produce structurally novel and diverse secondary metabolites [3,4,5], including polyketides, peptides, alkaloids, etc., numerous examples of which possess attractive cytotoxic, antitumor, antimicrobial, antifungal and anti-infective properties [6].

Remarkably, antibiotic resistance has emerged as an important global threat in recent years, reducing the possibility of curing diseases caused by various pathogens [7]. Therefore, the discovery of new compounds is encouraged in the fight against the threat posed by the increasing number of drug-resistant infectious diseases and upcoming disorders [8]. Due to the simple structure of sponges and the lack of an effective physical defense system, the metabolites with antibacterial activity produced by these associated microorganisms can help sponges to constitute a strong defense mechanism against competitors, predators and infectious microorganisms, which provide source molecules or innovative inspiration for the human development of new antibiotics.

Furthermore, many studies have paid more and more attention to whether the secondary metabolites of sponge-associated microorganisms have antibacterial activity in order to find lead compounds against multi-drug-resistant (MDR) strains. For example, Skariyachan et al. isolated many strains of symbiotic bacteria from Indian sponges in 2013 and 2015, respectively, and found that these strains could produce several types of secondary metabolites by screening methods and that these compounds were resistant to a variety of pathogenic bacteria, such as methicillin-resistant *Staphylococcus aureus* (MRSA), *Salmonella typhi*, *Klebsiella pneumoniae*, *Pseudomonas aeruginosa* and so on [9,10].

Although the secondary metabolites of sponge-associated microorganisms have been reviewed by many people, the number of compounds with antibacterial activity is very small and they have a single source. For example, Mayer et al. reviewed 19 compounds with antimicrobial activity derived from *Phylum Porifera* sponge-associated fungi [11]. Mehbub et al. reviewed 7 compounds with antimicrobial activity purified from *Porifera* sponge-associated fungi [12]. Jin et al. reviewed secondary metabolites of sponge-associated fungi, of which only 14 had antibacterial activity [13].

In this paper, we summarized 270 secondary metabolites isolated from sponge-associated microorganisms from 2012 to 2022, including terpenoids, polyketides, alkaloids, peptides, glucosides and so on. These compounds were obtained from different sources, which exhibited antimicrobial activities against *Staphylococcus aureus*, *Escherichia coli*, *Bacillus subtilis*, *Enterococcus faecalis*, *Vibrio parahaemolyticus*, *Mycobacterium tuberculosis*, etc. (Table 1). We hope that this review will shed light on the discovery of valuable secondary metabolites, with potential antibacterial activities from the sponge-associated microorganisms. 

## 2. Metabolite Sources, Structures and Activities

### 2.1. Marine Natural Products with Antimicrobial Activity from Sponge-Associated Fungi

Nowadays, marine-derived fungi are gaining increasing attention as a source of novel bioactive secondary metabolites [14] due to their high yield and relatively small molecular structure. Moreover, marine fungi associated with sponges are the center of attention of researchers and are known to produce an array of structurally varied secondary metabolites possessing potential biological properties [2].

#### 2.1.1. Terpenoids

**Figure 1:**
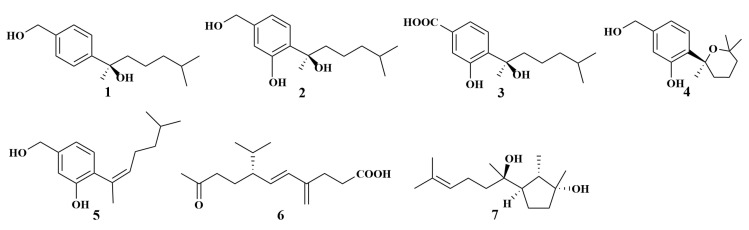
Structures of **1**–**7**.

*Aspergillus* sp. ZJ-2008004, derived from the *Xestospongia testudinaria* (South China Sea), produced four novel bisabolane-type sesquiterpenoids, aspergiterpenoid A (**1**), (−)-sydonol (**2**), (−)-sydonic acid (**3**) and (−)-5-(hydroxymethyl)-2-(2′,6′,6′-trimethyltetrahydro-2H-pyran-2-yl)phenol (**4**), as well as a known fungal metabolite (**5**). All compounds showed selective antibacterial activities against eight bacterial strains with minimum inhibitory concentration (MIC) values between 1.25 and 20.0 µg/mL [15]. *Fusarium* sp. KJMT. FP. 4. 3, isolated from the same genus sponge (Indonesian) as *Aspergillus* sp. ZJ-2008004, yielded two known compounds, tricinonoic acid (**6**) and cyclonerodiol (**7**) (Figure 1), which showed weakly antibacterial activities against multidrug-resistant *Salmonella enterica* ser. Typhi, with MIC values of 125 µg/mL for each [16].

**Figure 2:**
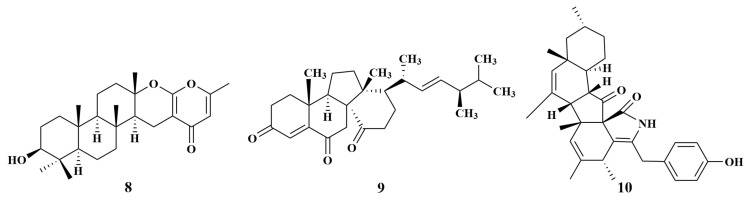
Structures of **8**–**10**.

Chevalone E (**8**), a novel chevalone derivative separated from *Aspergillus similanensis* KUFA0013 associated with *Rhabdermia* sp., showed moderate activity against MRSA [17]. A known compound, dankasterone A (**9**) from *Neosartorya fennelliae* KUFA0811, was associated with the sponge *Clathria reinwardtii* and displayed active activities against *S. aureus* and *Enterococcus faecalis*, with MIC values of 16, 32 µg/mL [18]. Diaporthalasin (**10**) (Figure 2), a new pentacyclic cytochalasin derivative, was collected from the *Phomopsis* sp. sponge-associated fungus *Diaporthaceae* sp. PSU-SP2/4. Bioassay results showed that **10** exhibited significant antibacterial activities against both *S. aureus* and MRSA, with equal MIC values of 2 µg/mL [19].

**Figure 3:**
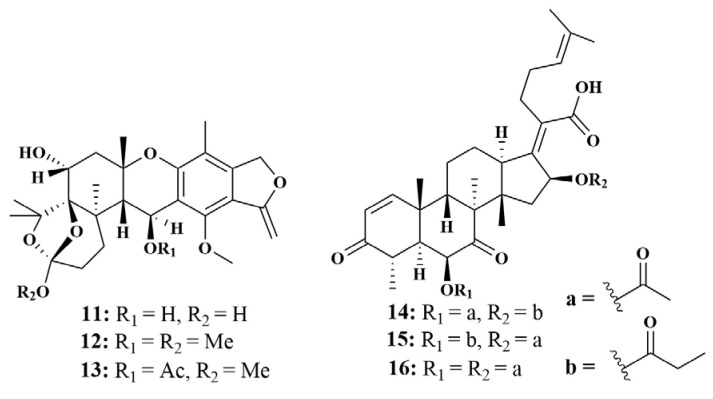
Structures of **11**–**16**.

A new meroterpenoid, austalide R (**11**), together with two known compounds, austalide M (**12**) and austalide N (**13**), were obtained from *Aspergillus* sp. from the Mediterranean sponge *Tethya aurantium*. Compounds **11** and **12** possessed broad-spectrum antibacterial activities against *Halomonas aquamarina*, *Polaribacter irgensii*, *Pseudoalteromonas elyakovii*, *Roseobacter litoralis*, *Shewanella putrefaciens*, *Vibrio harvey*, *V. natriegens*, *V. proteolyticus* and *Vibrio carchariae*, with MIC values of 0.001-10 µg/mL, while compound **13** can inhibit *H. aquamarine* and *V. natriegens*, with MIC values of 0.01 µg/mL, respectively [20]. Meanwhile, two novel helvolic acid derivatives, 16-*O*-propionyl-16-*O*-deacetylhelvolic acid (**14**) and 6-*O*-propionyl-6-*O*-dea-cetylhelvolic acid (**15**), and one known helvolic acid (**16**) (Figure 3) cultivated from an unidentified sponge-associated fungus, *A. fumigatus* HNMF0047 (Wenchang, China), both displayed stronger antibacterial activity against *Streptococcus agalactiae*, with MIC values of 16, 2 and 8 μg/mL, respectively [21].

**Figure 4:**
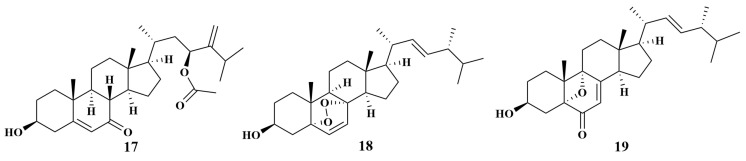
Structures of **17**–**19**.

*Acremonium* sp. NBUF150, collected from the *Ciocalypta* sponge (South China Sea), gave a new steroid, acremocholone (**17**), along with two known analogs, (22E)-5α,8α-epidioxyergo-sta-6,22-dien-3β-ol (**18**) and (22E,24R)-3β-hydroxy-5,9-epoxyergosta-7,22-dien-6-one (**19**) (Figure 4). Compound **17** exhibited antimicrobial inhibition against *V. scophthalmi*, *V. shilonii* and *V. brasiliensis*, with MIC values of 8 μg/mL, while **18** inhibited *V. shilonii* and *V. brasiliensis*, with MIC values of 8 and 32 μg/mL, respectively. In addition, compound **19** inhibited the growth of *V. brasiliensis*, with an MIC value of 16 μg/mL [22].

**Figure 5:**
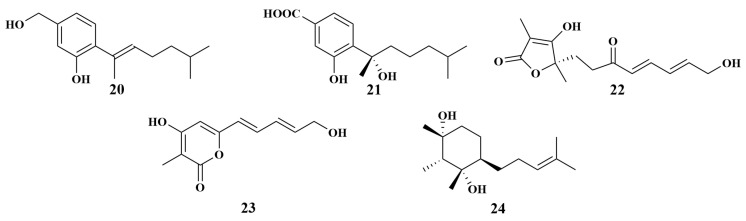
Structures of **20**–**24**.

An examination of the fungal strain *Aspergillus sydowii* ZSDS1-F6 (Xisha Islands, China) revealed aspergillusene A (**20**) and sydonic acid (**21**). Among them, **20** showed modest antimicrobial activities against *Klebsiella pneumonia* and *Aeromonas hydrophila*, with MIC values of 21.4 and 4.3 μM, respectively, while compound **21** can inhibit *Enterococcus faecalis*, with an MIC value of 18.8 μM [23]. Saturnispol F (**22**) and saturnispol H (**23**), two novel conjugated olefinic metabolites, were first isolated from marine-sponge-associated fungus *Trichoderma saturnisporum* DI-IA collected from the Xisha Islands, which provided additional evidence to support the assumption that the same fungal species from a marine environment are capable of activating a distinct biosynthetic pathway. Moreover, compounds **22** and **23** exerted selective effects against a panel of bacteria strains, including vancomycin-resistant *Enterococcus* (VRE) and *Bacillus subtilis*, with MIC values ranging from 1.63 to 12.9 µg/mL [24]. *Hypocrea koningii* PF04, also separated from the Xisha Islands sponge sample, produced citrantifidiol (**24**) (Figure 5), which displayed mild antibacterial activity against *Staphylococcus aureus*, with an MIC value of 32 μg/mL [25].

To find anti-*Vibrio harveyi* natural products, two *Haliclona* sp. sponge-associated fungal strains (Hainan, China), identified as *Aspergillus* sp. LS116 and *Penicillium* sp. LS54, produced aspergillsteroid A (**25**), neocyclocitrinol B (**26**) and penicillilactone A (**27**), respectively. The MIC values of the three compounds were 16, 128 and 8 μg/mL. Meanwhile, a comparison of the structures of **25** and **26** revealed that the absence of a C-23 hydroxyl branch moiety in **25** enhanced antibacterial activity. In addition, penicillilactone A (**27**) was the first example of a natural product containing a 7-membered lactone ring fused to a furan group [26,27].

**Figure 6:**
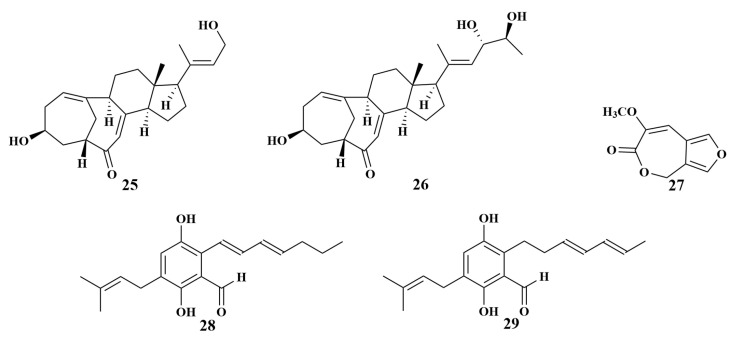
Structures of **25**–**29**.

Elena et al. reported the isolation and identification of two known compounds, dihydroauroglaucin (**28**) and isodihydroauroglaucin (**29**) (Figure 6), from *Eurotium chevalieri* MUT2316 associated with the Atlantic sponge *Grantia compressa*. Compound **28** showed inhibitory activities against *Staphylococcus aureus*, *Enterococcus faecalis* and *Streptococcus pneumoniae*, with MIC values of 124, 64 and 8 μg/mL, respectively, while compound **29** exhibited inhibitory activities against *S. aureus* and *E. faecalis*, with MIC values of 4–64 μg/mL [28].

**Figure 7:**
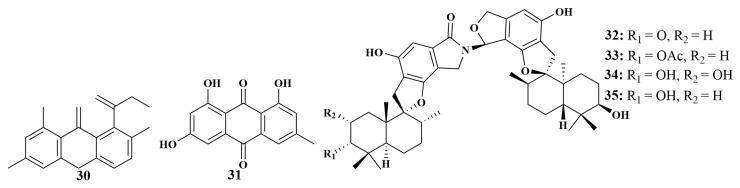
Structures of **30**–**35**.

Two known compounds, pinselin (**30**) and frangula-emodin (**31**), were produced by the strain of *Penicillium* sp. SCSIO41015 originating from the *Callyspongia* sp. sponge. They displayed weak antibacterial activity against *Staphylococcus aureus*, with MIC values of 57 and 3.75 µg/mL, respectively [29]. Further investigation led to the isolation of another four novel phenylspirodrimane-type dimers. Chartarlactams Q–S (**32**–**35**) (Figure 7) were discovered from *Stachybotrys chartarum* WGC-25C-6 associated with the *Niphates* sp. sponge (Beibu Gulf, China), and can also inhibit *S. aureus*, with MIC values in the range of 4–16 µg/mL [30].

#### 2.1.2. Polyketides

Chrysazin (**36**) and globosuxanthone A (**37**) were obtained from *Beauveria bassiana* TPU942 cultivated from an unidentified sponge (Iriomote Island, Okinawa), which showed an inhibition zone of 7 mm against *Candida albicans* at a concentration of 10.0 μg/disk [31]. Four known compounds cordyol A (**38**), diorcinol (**39**), isochaetochromin B_2_ (**40**) and ustilaginoidin D (**41**) were isolated from *Megatherium anisopliae* MXH-99 (Weizhou Island, China). Compounds **38** and **39** were reported to have weak antimycobacterial activities against *Mycobacterium tuberculosis*, with MIC values of 100.0 and 50.0 µg/mL, respectively [32]. In addition, isochaetochromin B_2_ (**40**) and ustilaginoidin D (**41**) exhibited the highest activities against *Mycobacterium phlei*, with MIC values of 50.0 µg/mL for each [33].

**Figure 8:**
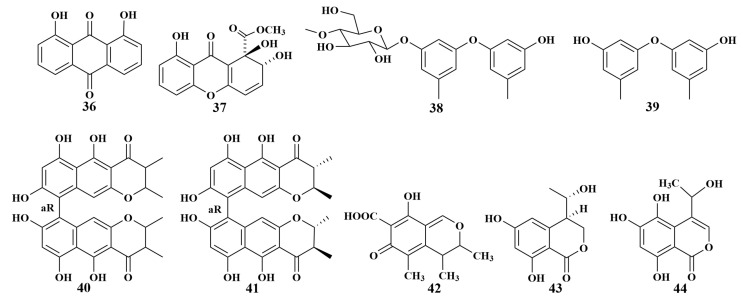
Structures of **36**–**44**.

The isolation of citrinin (**42**) was reported in 2013 from *Penicillium* sp. FF001 associated with the sponge *Melophlus* sp., which exhibited potent antibacterial activities against MRSA, rifampicin-resistant *Staphylococcus aureus*, wild-type *Staphylococcus aureus* and vancomycin-resistant *Enterococcus faecium*, with MIC values of 3.90, 0.97, 1.95 and 7.81 µg/mL, respectively. Further, it also displayed significant activity against *Cryptococcus neoformans*, with an MIC value of 3.90 µg/mL [34]. Penicifurans A (**43**) and 5, 6, 8-trihydroxy-4-(1′-hydroxyethyl)-isocoumarin (**44**) (Figure 8) were isolated from *Penicillium* sp. MWZ14-4 (South China Sea). Among these, compound **43** showed moderate inhibitory activity against *Streptomyces albus*, with an MIC value of 3.13 μM, and weak activity against *Bacillus cereus*, and compound **44** displayed mild activities against *Bacillus cereus* and *Vibrio parahemolyticus*, with MIC values of 6.25 μM for each [35].

Helicusin A (**45**), deacetylsclerotiorin (**46**) and isochromophilone XI (**47**) were produced from the culture of the *Tethya aurantium* sponge-associated fungus *Bartalinia robillardoides* LF550 (Mediterranean), which is the first report on the isolation of chloroazaphilones of a fungal strain belonging to the genus *Bartalinia*. Remarkably, weak antibacterial activities against *Bacillus subtilis* and *Staphylococcus lentus* were found in compounds 46 and 47, and a significant inhibition of *Candida albicans*, *Trichophyton rubrum* and *Septoria tritici* was found in compounds 45 and **46**, while **47** revealed specifically weak activity against *T. rubrum* [36]. In continuing the search for biologically active secondary metabolites in Mediterranean sponge-associated fungi, *Talaromyces* sp. LF458 afforded two novel oxaphenalenone dimers, talaromycesone A (**48**) and talaromycesone B (**49**), together with one known diphenyl ether derivative AS-186c (**50**), which displayed strong antimicrobial activities against *S. epidermidis*, with half-inhibitory concentration (IC_50_) values of 3.7, 17.36 and 1.24 μM, and MRSA, with IC_50_ values of 5.48, 19.5 and 1.71 μM, respectively [37].

**Figure 9:**
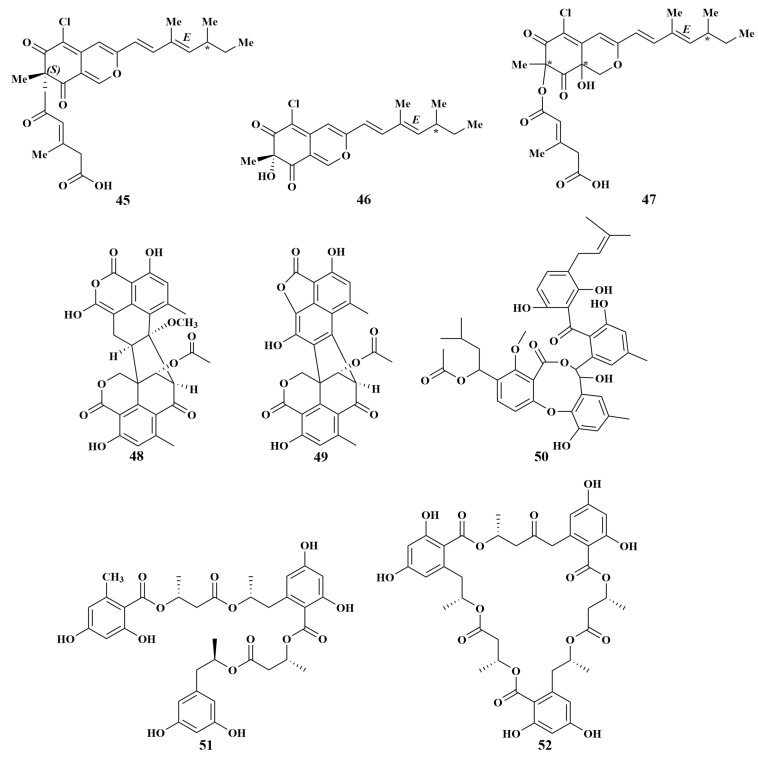
Structures of **45**–**52**. (* represents configulation undetermined).

*Hansfordia sinuosae*, recovered from the *Niphates* sp. sponge (South China Sea), was the source of a new polyester, hansforester A (**51**), and a known analogue, ascotrichalactone A (**52**) (Figure 9). Both **51** and **52** exhibited potent inhibition against a panel of bacterial strains, including the agricultural pathogenic bacteria, *Pseudomonas lachrymans*, *Agrobacterium tumefaciens*, *Xanthomonas vesicatoria* and *Ralstonia solanacearum*, with MIC values of 15.6 μM for each, and the human-infected bacterium *Staphylococcus aureus*, with MIC values of 3.9 μM for each [38].

In 2017, Lei Hui et al. reported the isolation and identification of two novel isocoumarins, pestaloisocoumarins A (**53**) and B (**54**), together with one known isocoumarin, gamahorin (**55**), and three chlorinated benzophenone derivatives, pestalachlorides B (**56**) and E (**57**) and a mixture of pestalalactone atropisomers (**58a**/**58b**), from *Pestalotiopsis heterocornis* XWS03F09 associated with *Phakellia fusca* collected at Xisha Islands of China. Additionally, six undescribed polyketide derivatives, heterocornols A (**59**), heterocornols B (**60**), heterocornols C (**61**), heterocornols F (**62**), heterocornols G (**63**) and heterocornols H, (**64**) and their known analogues, methyl-(2-formyl-3-hydroxyphenyl)p-ropanoate (**65**), agropyrenol (**66**) and vaccinol G (**67**), were also discovered from this strain. All compounds showed antibacterial activities against *Staphylococcus aureus* and *Bacillus subtilis*, with MIC values ranging from 3 to 100 µg/mL [39,40]. Moreover, compounds **53**, **54**, **55**, **61**, **63**, **66** and **67** showed weak antifungal activities against *Candida parapsilosis* and *Cryptococcus neoformans*, with MIC values of 100 µg/mL for each. To further explore more diverse polyketides, karimunones B (**68**), rhodolamprometrin (**69**), 7-*O*-methylrhodolam-prometrin (**70**) and 6-*O*-methylSMA93 (**71**) were isolated from the same fungal strain *Fusarium* sp. KJMT. FP. 4. 3 as tricinonoic acid (**6**), and had the same antibacterial activities [16].

**Figure 10:**
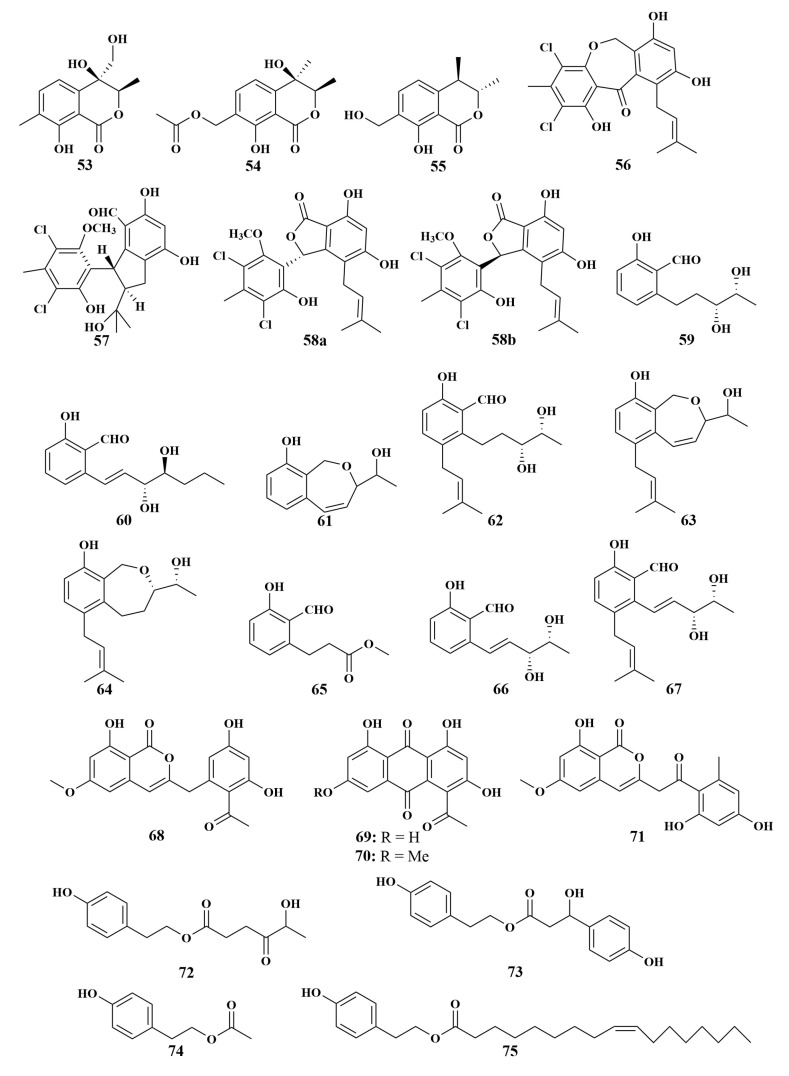
Structures of **53**–**75**.

Meanwhile, to further explore more *Phakellia fusca* sponge-associated fungi in Xisha Islands, *Hypocrea koningii* PF04 resulted in the discovery of hypocrol A (**72**), trichodenol B (**73**), 4-hydroxyphenethyl acetate (**74**), and 1-oleyltyrosol (**75**) (Figure 10). Both of them possessed weak antibacterial activities against *Staphylococcus aureus*, *Escherichia coli* and MRSA, with MIC values in the range of 64–128 µg/mL [41]. 

Three new heterdimeric tetrahydroxanthone-chromanone lactones, chrysoxanthones A–C (**76**–**78**), were found in *Penicillium chrysogenum* HLS111 obtained from the sponge *Gelliodes carnosa*. The bioassay test indicated that compounds **76**–**78** exhibited the highest antibacterial activity against *Bacillus subtilis*, with MIC values of 5–10 µg/mL, and moderate activities against *Staphylococcus epidermidis* and *S. aureus*, with MIC values of 10-80 µg/mL, respectively [42]. Penicitrinone A (**79**) and penicitrinol J (**80**) were reported in 2020 from *Penicillium citrinum* WK-P9 associated with the *Suberea* sp. sponge (Hoga Island). Compound **79** showed moderate activity against *Mycobacterium smegmatis*, with an MIC value of 32 µg/mL. Compound **80** exhibited moderate growth inhibition against *Bacillus subtilis*, *B. megaterium* and *M. smegmatis*, with MIC values of 16, 16 and 32 µg/mL, respectively. Furthermore, a weak activity of 64 µg/mL against *S. aureus* was observed for **80** [43]. *Penicillium vinaceum* isolated from the sponge *Hyrtios erectus* (Yanbu) yielded citreoisocoumarin (**81**) and showed activity against *S. aureus*, with an inhibition zone of 19 mM [44].

*Aspergillus versicolor* MF359, obtained from the sponge *Hymeniacidon perleve* (Bohai Sea, China), led to the isolation of a new secondary metabolite, 5-methoxydihydrosterigmatocystin (**82**), which displayed effective activities against *Bacillus subtilis* and *Staphylococcus aureus*, with MIC values of 3.13 and 12.5 μg/mL, respectively [45]. In a further chemical investigation of the same genus sponge in the Bohai Sea, China, two known compounds, emodin (**83**) and trypacidin (**84**), were purified from the culture broth of *Aspergillus fumigatus* MF029. All of them displayed obvious antibacterial activities against MRSA and *S. aureu*, with MIC values of 50 µg/mL, and significant activity against BCG, with MIC values of 1.25 µg/mL. Moreover, trypacidin (**84**) showed effective activity against *Bacillus subtilis*, with MIC values of 12.5 µg/mL. This was the first report of trypacidin (**84**) displaying potential antitubercular activity, which would promote the investigation of antitubercular secondary metabolites from fungi [46].

**Figure 11:**
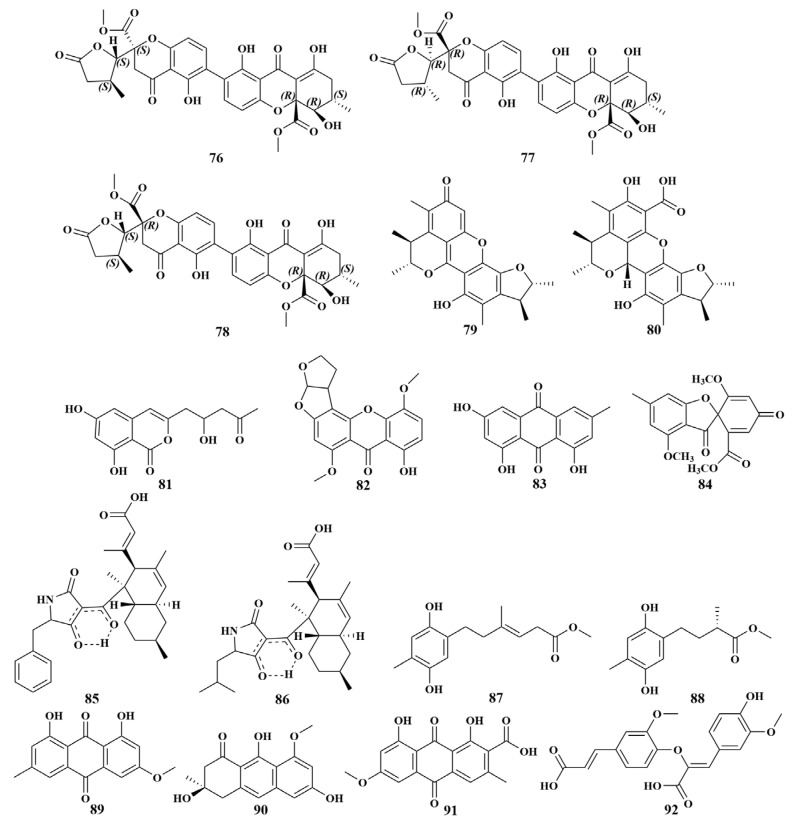
Structures of **76**–**92**.

An unusual polyketide with a new carbon skeleton, lindgomycin (**85**), and the recently described ascosetin (**86**), were extracted from the culture broth of *Lindgomycetaceae* sp. KF970 and *Lindgomycetaceae* sp. LF327 (Baltic Sea, Antarctic), respectively. Compounds **85** and **86** exhibited extensive antibiotic activities against *Staphylococcus epidermidis*, *Staphylococcus aureus*, MRSA, *Propionibacterium acnes*, *Candida albicans*, *Xanthomonas campestris* and *Septoria tritici*, with IC_50_ values in the range of 2–18 µM [47]. *Penicillium* sp. HDN151272 also derived from an Antarctica sponge produced two new polyketides, ketidocillinones B (**87**) and ketidocillinones C (**88**). Bioassay results showed that these two compounds possessed selective and broad-spectrum antibacterial activities against *Mycobactrium phlei*, *Pseudomonas aeruginosa*, meticillin-resistant coagulase-negative *Staphylococcus* (MRCNS), *B. subtilis*, *B. cereus* and *V. parahemolyticus*, with MIC values ranging from 1.56 to 25.00 μg/mL [48]. In order to explore more compounds with broad-spectrum antibacterial activities, physcion (**89**), asperflavin (**90**) and cinnalutein (**91**) were isolated from the same fungal strain *Eurotium chevalieri* MUT2316 as compounds **28** and **29** [28]. In addition to the above compounds, 8-*O*-4-dehydrodiferulic acid (**92**) (Figure 11) was also able to inhibit a range of pathogenic bacteria [20].

**Figure 12:**
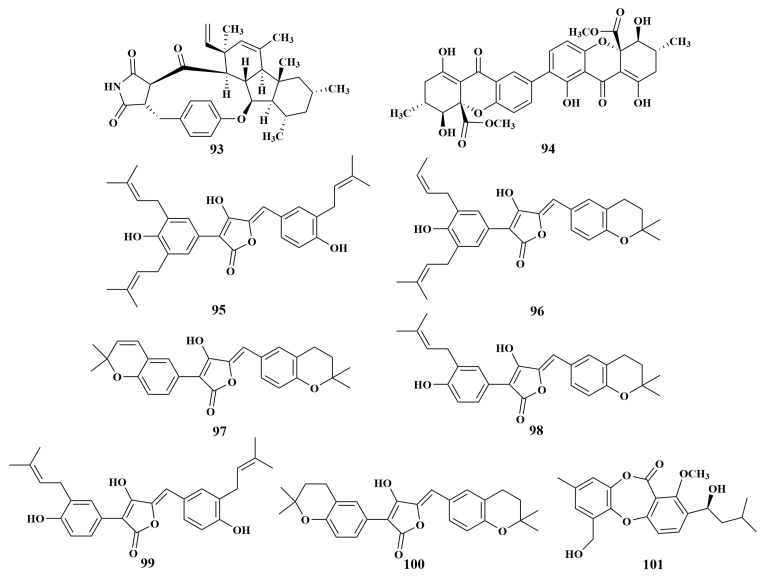
Structures of **93**–**101**.

GKK1032B (**93**) and secalonic acid A (**94**) were purified from *Penicillium erubescens* KUFA0220 associated with the sponge *Neopetrosia* sp., which acts against *Enterococcus faecalis*, VRE *Enterococcus faecium* and *Staphylococcus aureus*, with MIC values of 8, 8 and 32 μg/mL, respectively, while secalonic acid A (**94**) acted against MRSA with an MIC value of 64 µg/mL [49]. Except for **93** and **94**, aspulvinones R (**95**), aspulvinones S (**96**) and aspulvinones U (**97**), along with three known compounds aspulvinones B′ (**98**), aspulvinones H (**99**) and aspulvinones A (**100**) from *Aspergillus flavipes* KUFA1152 associated with the Thailand sponge *Mycale* sp., had the same antimicrobial activities, with MIC values ranging from 4 to 16 µg/mL [50]. Further investigation led to the isolation of another novel compound, spinolactone (**101**) (Figure 12), which was separated from *N. spinosa* KUFA1047 associated with the same genus sponge as **95**–**100** and displayed antibacterial activity against *E. faecalis*, with an MIC value of 64 µg/mL [51].

*Aspergillus carneus*, recovered from the sponge *Agelas Oroides* (Aegean Sea, Turkey), was the source of 5′-epi-averufanin (**102**), versicolorin C (**103**) and averufin (**104**). Among them, compound **102** was found to be active against *Staphylococcus aureus* and *Enterococcus faecium*, with MIC values of 4.6 and 9.3 μg/mL, respectively, and compound **103** showed inhibitory activity against *S. aureus*, with an MIC value of 4.3 μg/mL. Moreover, compound **104** exhibited inhibitory activities against *S. aureus*, *E. faecium* and *E. faecalis*, with MIC values of 4.6, 2.3 and 18.4 μg/mL, respectively. This study used the “One Strain Many Compounds” (OSMAC) culture strategy, which broke the tradition of using only one fermentation method for most studies on fungal natural products [52]. In the continuing search for biologically active compounds in Turkish sponge-associated fungi, *Rhizopus oryzae* was afforded three new mycophenolic acid derivatives, penicacids H–J (**105**–**107**), together with two known naphtho-γ-pyrone dimers, asperpyrone A (**108**) and dianhydroaurasperone C (**109**), which acted against *S. aureus*, MRSA, *Escherichia coli* and *Pseudomonas aeruginosa*, with MIC values in the range of 62.5–250 μg/mL [53].

**Figure 13:**
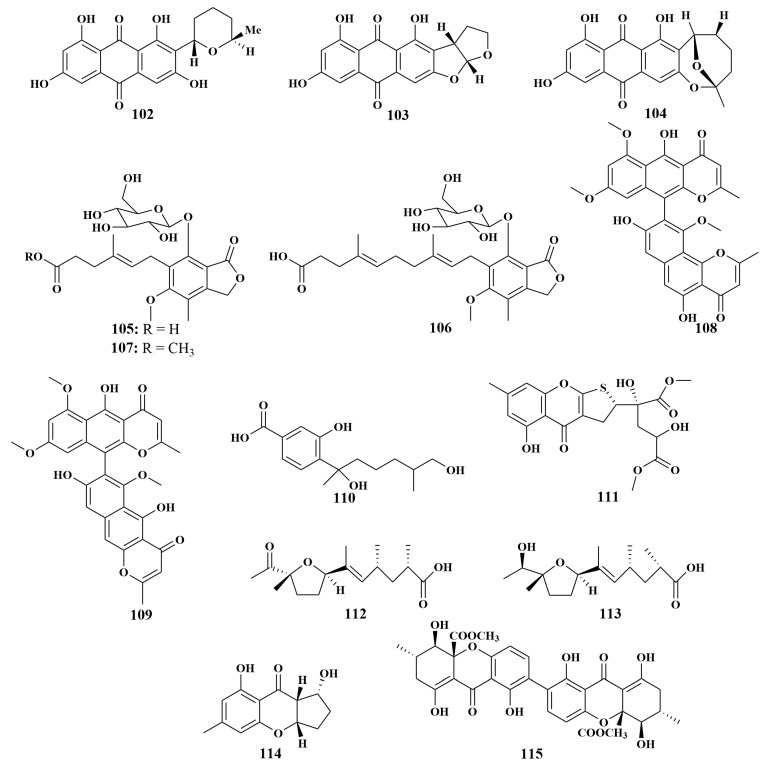
Structures of **102**–**115**.

Li et al. reported the isolation of two known compounds, 12-hydroxysydonic acid (**110**) and oxalicumone A (**111**), in 2019, from *Aspergillus* sp. LS34 obtained from the sponge *Haliclona* sp. (Hainan, China). Compound **110** had important inhibitory activity against *Staphylococcus aureus*, with an MIC value of 3.54 μM, while compound **111** showed weak inhibitory activity against *Escherichia coli*, with an MIC value of 75.4 μM [54]. Another two fungal strains, *Aspergillus* sp. LS78 and *Aspergillus* sp. LS57, also originated from the *Haliclona* sp. sponge in Hainan, yielding aspericacids A (**112**) and B (**113**), which contain an unusual 2,5-disubstituted tetrahydrofuran ring and unsaturated fatty acid chain, as well as aspergilluone A (**114**). Compound **112** possessed moderate inhibitory activities, with MIC values of 50 μg/mL against both *Candida albicans* and *Cryptoccus neoformans*, while compound **113** displayed slightly weak activity, with MIC values of 128 μg/mL. Compound **114** showed selective antibacterial activities against *Mycobacterium tuberculosis*, *Staphylococcus aureus*, *Bacillus subtilis* and *Escherichia coli*, with MIC values of 32–128 μg/mL [55,56]. Secalonic acid D (**115**) (Figure 13) derived from *Aspergillus* sp. SCSIO XWS03F03 exhibited mild antimicrobial activities against *Staphyloccocus aureus* and *Mycobacterium tuberculosis*, with IC_50_ values of 7.19 and 1.26 μM, respectively [57].

**Figure 14:**
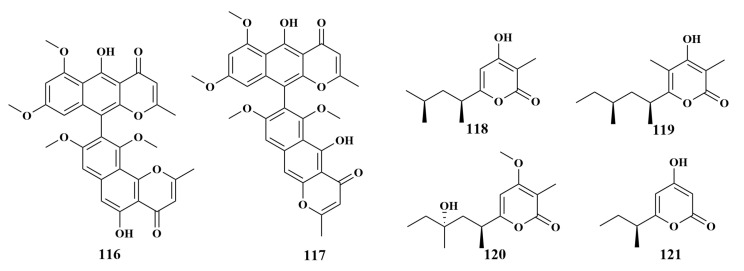
Structures of **116**–**121**.

Fonsecinone A (**116**) and isoaurasperone A (**117**), discovered from *Reniera japonica*-associated fungus *Aspergillus niger* L14, was the first report possessing potent anti-*Helicobacter pylori* activity for dimeric naphtho-γ-pyrones, with MIC values of 2–4 µg/mL [58]. *Aspergillus niger* LS24, isolated from the same genus sponge as *Aspergillus niger* L14, gave three novel 4-hydroxy-α-pyrones, nipyrones A-C (**118**–**120**), together with one known compound, germicidin C (**121**) (Figure 14). Compound **120** exhibited significant inhibitory activities against *Staphylococcus aureus* and *Bacillus subtilis*, with MIC values of 8 and 16 µg/mL. Compounds **118**, **119** and **121** exhibited moderate antimicrobial effects against *S. aureus*, *Escherichia coli* and *B. subtilis*, with MIC values in the range of 32–64 µg/mL. Significantly, **118**–**121** showed weak activity against MRSA, with MIC values of 128 µg/mL for each [59].

A new anthraquinone, versiconol B (**122**), along with two known polyketides, versiconol (**123**) and sterigmatocystin C (**124**), were purified from the culture broth of *Aspergillus* sp. F40, which was associated with *Callyspongia* sp. (Xuwen Country, China). Both of them were able to selectively inhibit *Staphylococcus aureus* and *Vibrio parahaemolyticus*, with MIC values ranging from 12 to 48 µg/mL [60]. Another two fungal strains, *Alternaria* sp. SCSIO41014 and *Fusarium equiseti* SCSIO41019, were also collected from the *Callyspongia* sp. sponge in Xuwen Country, producing alterlactone (**125**) and linoleicacid (**126**), respectively. Alterlactone(**125**) showed mild inhibitory activity against *Staphylococcus aureus*, with an MIC value of 31.25 µg/mL. Linoleicacid (**126**) possessed antibacterial activities against *S. aureus* and MRSA, with MIC values ranging from 2 to 125 μg/mL [61,62].

**Figure 15:**
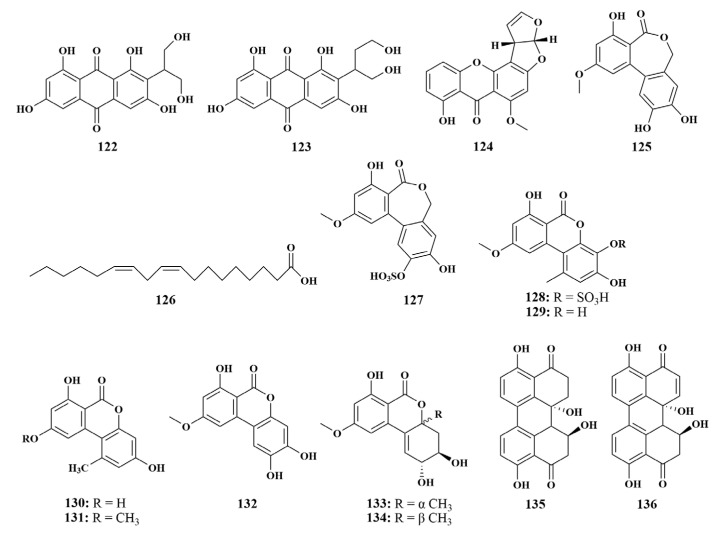
Structures of **122**–**136**.

In the continuing search for biologically active secondary metabolites in *Callyspongia* sp. sponge-associated fungi (Xuwen Country, China), two new dibenzopyrones with a rare sulfate group, alterlactone 5′-*O*-sulfate (**127**) and 3′-hydroxyalternariol-5-*O*-methyl ether-3′-*O*-sulfate (**128**), as well as eight known compounds 5-*O*-methyl ether (**129**), alternariol (**130**), alternariol-5-*O*-methyl ether (**131**), altenusiol (**132**), isoaltenuene (**133**), altenuene (**134**), dihydroalterperylenol (**135**) and alterperylenol (**136**) (Figure 15) were discovered. All compounds showed mild inhibitory activities and a broad spectrum against eight foodborne bacteria, with MIC values ranging from 15.63 to 125 μg/mL [63].

**Figure 16:**
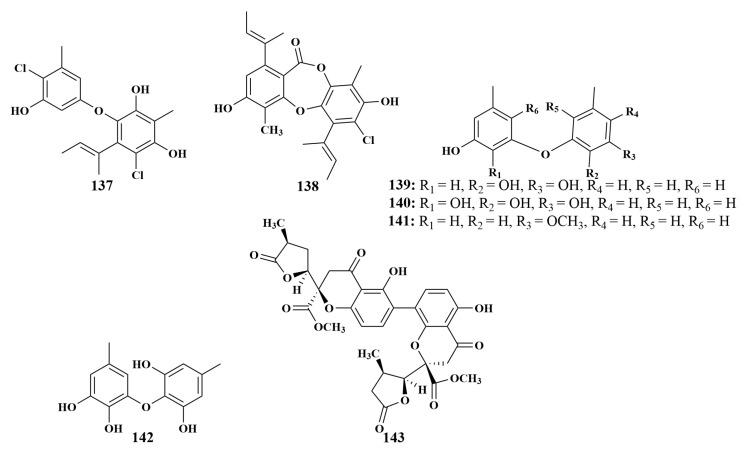
Structures of **137**–**143**.

A new diphenyl ether, aspergillusether E (**137**), was given by the fungus *Aspergillus unguis* PSU-MF16 associated with *Dysidea* sp. (Thailand). Additionally, the previously known emeguisin A (**138**) was also discovered from the same strain. Compound **137** displayed the best antimicrobial activities against *Staphylococcus aureus* and MRSA, with equal MIC values of 16 μg/mL. Compound **138** displayed potent antimicrobial activities against *S. aureus*, MRSA and *Cryptococcus neoformans*, with equal MIC values of 0.5 μg/mL [64]. Four known compounds cordyol C (**139**), violaceol II (**140**), violaceol I (**141**) and cordyol E (**142**) were isolated from *Aspergillus sydowii* J05B-7F-4 derived from the sponge *Stelletta* sp., which showed mild antibacterial activities against *S. aureus*, *Streptococcus iniae* and *Vibrio ichthyoenteri* [65]. Paecilin E (**143**) (Figure 16) was obtained from the same fungal strain *Neosartorya fennelliae* KUFA0811 as dankasterone A (**9**), which was active against *S. aureus* and *Enterococcus faecalis*, with MIC values of 16 and 32 μg/mL, respectively [18].

#### 2.1.3. Alkaloids

In an examination of the fungal strain *Stagonosporopsis cucurbitacearum*, two novel 4-hydroxy-2-pyridone alkaloids containing hydroxamic acid moiety, didymellamides A (**144**) and B (**145**), were discovered. Compound **144** inhibited the growth of *Candida albicans*, *C. glabrata* and *Cryptococcus neoformans* at concentrations of 1.6 or 3.1 μg/mL, whereas **145** inhibited only *C. neoformans*, with an MIC value of 6.3 μg/mL [66]. Equisetin (**146**) and 5′-epiequisetin (**147**) were isolated from the same fungal strain *Fusarium* sp. KJMT. FP. 4. 3 as linoleicacid (**126**), and had the same antibacterial activities [62]. *Fusarium* sp. LY019 derived from *Suberea mollis* led to the identification of fusaripyridines A (**148**) and B (**149**), which were the first examples of natural products possessing a 1,4-bis(2-hydroxy-1,2-dihydropyridin-2-yl)butane-2,3-dione backbone. These two compounds selectively inhibited the growth of *C. albicans*, with MIC values down to 8.0 µM, while they were moderately active against *Staphylococcus aureus* and *Escherichia coli*, with the diameters of inhibition circle ranging from 7 to 9 mM [67].

**Figure 17:**
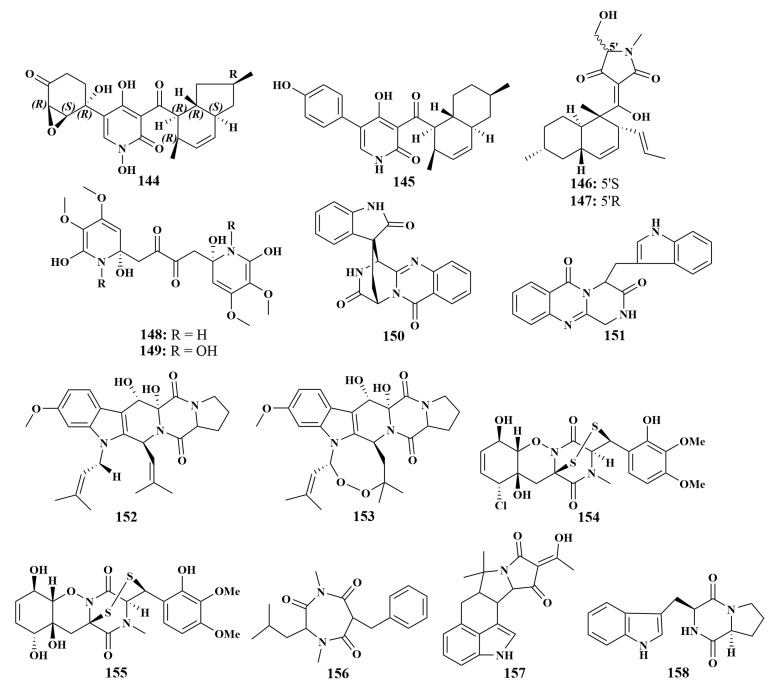
Structures of **144**–**158**.

The sponge-associated fungi *Penicillium adametzioides* AS-53 (Hainan, China) led to the discovery of lapatins B (**150**), glyantrypine (**151**), fumitremorgin B (**152**) and verruculogen (**153**). All compounds showed extensive inhibitory activity against the aqua-bacterial *Vibrio harveyi*, with MIC values of 16.0, 32.0, 32.0 and 32.0 μg/mL, respectively [68]. Further chemical investigation of this strain led to another two important compounds, adametizines A (**154**) and B (**155**). Compound **154** was found to be active against *Staphylococcus aureus*, *Aeromonas hydrophilia*, *V. harveyi*, *Vibrio parahaemolyticus* and *Gaeumannomyces graminis*, with MIC values of 8, 8, 32, 8 and 16 μg/mL, respectively, whereas **155** only showed activity against *S. aureus*, with an MIC value of 64 μg/mL [69]. These data indicate that the C-l substitution at C-7 significantly increased the brine shrimp lethality and antimicrobial activity. Terretrione A (**156**), α-cyclopiazonic acid (**157**) and brevianamide F (**158**) (Figure 17) were obtained from the same fungus as **81**, which showed selective effects against *S. aureus*, *Escherichia coli* and *Candida albicans*, with inhibition zones of 19–27 mM [44].

Besides compounds **11**, **12**, **13** and **92**, *Aspergillus* sp. was also the source of 3-((1-hydroxy-3-(2-methylbut-3-en-2-yl)-2-oxoindolin-3-yl)methyl)-1-methyl-3,4-dihydrobenzo[e][1,4]diazepine-2,5-dione (**159**), dihydroisoflavipucine (**160**) and cytochalasin Z17 (**161**), and had the same antibacterial activities [20]. Wang et al. reported the isolation and identification of one known compound, notoamide F (**162**), from *Aspergillus sclerotiorum* GDST-2013-0501 (South China Sea) in 2022, which showed moderate antibacterial activity against *Staphylococcus epidermidis*, with an MIC value of 12.5 µM [70]. Preussin (**163**), purified from *Aspergillus candidus* KUFA0062 associated with the sponge *Epipolasis* sp., exhibited broad antibacterial activities against *Staphylococcus aureus*, *Enterococcus faecalis*, VRE *E. faecalis* and MRSA, with equal MIC values of 32 µg/mL. Remarkably, it was the first report of the isolation of hydroxypyrrolidine alkaloids from *A. candidus*, and the presence of N-methyl on the cloalkane ring of this compound was important for its antibacterial activities. It also had the property of broadly inhibiting pathogenic bacteria and interfering with the biofilm formation of pathogenic strains, and thus could be used for the development of new antibiotics and anticancer drugs [71]. Perinadines B (**164**) and C (**165**) exhibited moderate in vitro antibacterial activity against *Bacillus subtilis*, with MIC values of 32 and 64 μg/mL, respectively. In addition, compared with secondary metabolites of *Aspergillus* sp., this class of tetracyclic skeleton alkaloids was rarely found in *Aspergillus* sp. LS116 [72].

**Figure 18:**
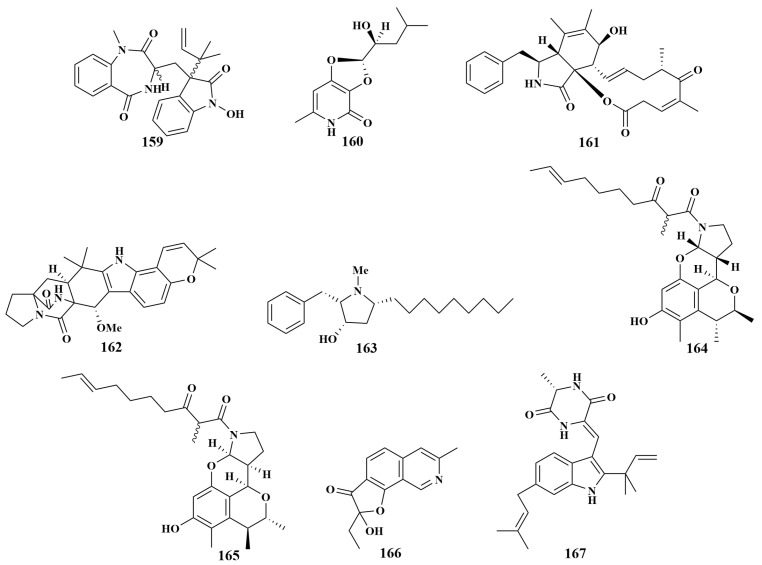
Structures of **159**–**167**.

Li Wei et al. separated a known compound (**166**) from *Penicillium chrysogenum* LS16, cultivated from an unidentified sponge (Hainan, China). This compound can strongly inhibit *Vibrio parahaemolyticus*, with an inhibition circle diameter of 28.36 mM [73]. Neoechinulin D (**167**) (Figure 18) was isolated from the same fungal strain *Eurotium chevalieri* MUT2316 and had the same antimicrobial activities as **28**, **29** [28].

#### 2.1.4. Peptides

Guided by anti-*Staphylococcus epidermidis*, strain *Penicillium* sp. F37 associated with the sponge *Axinella corrugate* (South Brazilian Coast) was continued for a further chemical investigation and led to cis-cyclov(Leucyl-Tyrosyl) (**168**), which showed strong antibiotic activity, with an 85% inhibition rate. This is the first time that compound **168** has demonstrated antibacterial activity and it could be a promising antimicrobial lead compound [74]. Two new peptides, peniciadametizine A (**169**) and B (**170**), were discovered from *Penicillium adametzioides* AS-53, which was derived from an unidentified sponge (Hainan, China). Both 169 and 170 demonstrated antibacterial activities against *Alternaria brassicae*, with MIC values of 4.0 and 32.0 µg/mL, respectively [75].

**Figure 19:**
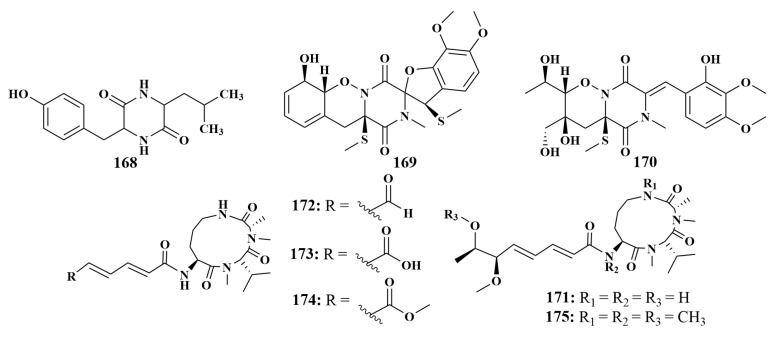
Structures of **168**–**175**.

Two known analogues sclerotiotides L (**171**) and F (**172**), together with three new aspochracin-type cyclic tripeptides sclerotiotides M–O (**173**–**175**) (Figure 19), were produced by *Aspergillus insulicola* HDN151418, obtained from an unidentified sponge (Prydz Bay, Antarctica). Compounds **173** and **174** showed broad antimicrobial activities against a panel of pathogenic strains, including *Bacillus cereus*, *Proteus* species, *Mycobacterium phlei*, *Bacillus subtilis*, *Vibrio parahemolyticus*, *Edwardsiella tarda*, MRCNS and MRSA, with MIC values ranging from 1.56 to 25 µM, while compounds **171**, **172** and **175** were less active [76].

**Figure 20:**
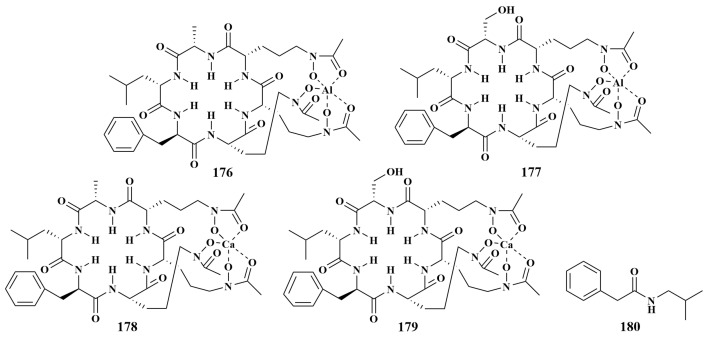
Structures of **176**–**180**.

The isolation of four new siderophore analogues chelating gallium ions and aluminum ions, Al(III)-acremonpeptide E (**176**), Ga(III)-acremonpeptide E (**177**), Al(III)-acremonpeptide F (**178**) and Ga(III)-acremonpeptide F (**179**), was reported in 2021 from *Acremonium persicinum* F10 obtained from marine sponge *Phakellia fusca* in the South China Sea. Compounds **176**–**179** displayed obvious antifungal activities against *Aspergillus fumigatus* and *Aspergillus niger*, with MIC values ranging from 1 to 3 µM [77]. Further chemical investigation of the same sponge *Phakellia fusca* in the South China Sea led to the discovery of N-isobutyl-2-phenylacetamide (**180**) (Figure 20) from the same fungal strain as citrantifidiol (**24**), which showed mild antibacterial activities against *Staphylococcus aureus*, with an MIC value of 32 μg/mL [25].

#### 2.1.5. Others

Besides compounds **24** and **180**, *Hypocrea koningii* PF04 also yielded a new furan derivative hypofurans A (**181**). Moreover, the same antimicrobial activity of 32 µg/mL against *Staphylococcus aureus* was also observed for compound **181** [25]. In the continuing search for biologically active compounds in *Phakellia fusca* sponge-associated fungi, tetradecanoate (**182**) was recovered from the same fungus as compounds **72**–**75**, and had the same antibacterial activities [41].

**Figure 21:**
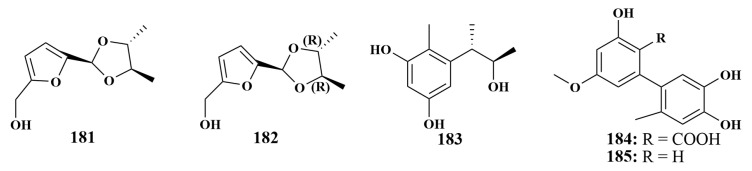
Structures of compounds **181**–**185**.

Phenol A (**183**) was produced by the strain of *Penicillium* sp. SCSIO41015 derived from the *Callyspongia* sp. sponge, which showed weak antibacterial activity against *Acinetobacter baumannii*, with an MIC value of 57 µg/mL [29]. Altenusin (**184**) and 5′-methoxy-6-methyl-biphenyl-3,4,3′-triol (**185**) (Figure 21) had broad-spectrum antibacterial activities that were the same as compounds **127**–**136** [63].

### 2.2. Marine Natural Products with Antimicrobial Activity from Sponge-Associated Actinomycetes

Marine actinomycetes are one of the most important resources for the mining of new natural products. The sponge-associated actinomycetes are not only rich and diverse, but also produce structurally novel secondary metabolites, including mainly polyketides, alkaloids, fatty acids, peptides and terpenoids, which have biological activities such as antibacterial, antitumor and antiparasitic activities [78].

#### 2.2.1. Polyketides

The isolation of urdamycinones E (**186**), urdamycinones G (**187**) and dehydroxyaquayam-ycin (**188**) was reported in 2012 from *Streptomycetes* sp. BCC45596 associated with the *Xestospongia* sp. sponge collected from Tianland. All compounds strongly inhibited *Mycobacterium tuberculosis*, with MIC values ranging from 3.13 to 12.5 µg/mL [79].

**Figure 22:**
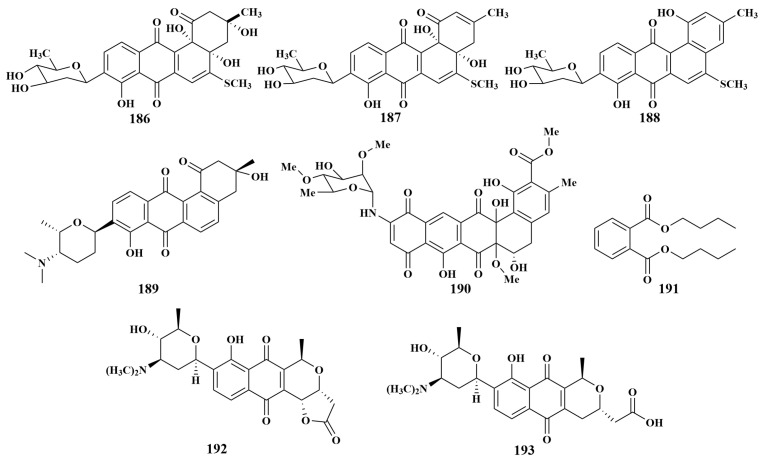
Structures of **186**–**193**.

*Streptomyces* sp. M7-15 was separated from the marine sponge *Scopalina ruetzleri* (Mona Island), from which frigocyclinone (**189**) was discovered, which exhibited weak antibiotic activity against *Bacillus subtilis* [80]. In addition, frigocyclinone (**189**) showed an inhibition zone of 1 mM at a concentration of 1 mg/mL against *Mycobacterium smegmatis* [81]. A naphthacene glycoside, SF2446A2 (**190**), was extracted from the culture broth of *Streptomyces* sp. RV15 associated with Mediterranean sponge *Dysidea tupha*, and showed inhibitory activity against *Chlamydia trachomatis* [82]. Dibutylphthalate (**191**) was purified from *Streptomyces* sp. LS298, associated with the *Fleshy knotted* sponge (South China Sea), which can inhibit *B. subtilis*, with an inhibition circle diameter of 8 mM [83]. *Streptomyces albolongus* CA-186053, also obtained from the same genus actinomycete as the above compounds, produced medermycin (**192**) and antibiotic G15-F (**193**) (Figure 22). These two compounds were able to inhibit MRSA, with MIC values of 2 and 4 µg/mL, respectively, and had weak inhibitory activity against *Escherichia coli*, with MIC values of 32–64 µg/mL. This article constituted the first report on the chemical composition of extracts from a marine-derived *Streptomyces* strain related to *S. albolongus*, and identified this strain as a new source of medermycins [84].

Additionally, in order to further explore more *Streptomyces* sp. actinomycetes, *Streptomyces coelicolor* LY001 resulted in the discovery of three new natural chlorinated derivatives of 3-phenylpropanoic acid, 3-(3,5-dichloro-4-hydroxyphenyl)propanoic acid (**194**), 3-(3,5-dichloro-4-hydroxyphenyl)propanoic acid methyl ester (**195**) and 3-(3-chloro-4-hydroxyphenyl)propanoic acid (**196**), along with 3-phenylpropanoic acid (**197**). Both of them inhibited *Staphylococcus aureus*, *E.coli* and *Candida albicans*, with inhibition circles of 7 to 23 mM in diameter [85].

Three new lavandulylated flavonoids, (2S,2″S)-6-lavandulyl-7,4′-dimethoxy-5,2′-dihydroxylflavanone (**198**), (2S,2″S)-6-lavandulyl-5,7,2′,4′-tetrahydroxylflavanone (**199**) and (2″S)-5′-lavandulyl-2′-methoxy-2,4-4′,6′-tetrahydroxylchalconev (**200**), together with two known compounds, (2S,2″S)-6-lavandulyl-7-methoxy-5,2′,4′-trihydroxylflavanone (**201**) and 6-prenyl-4′-methoxy-5,7-dihydroxylflavanone (**202**), were the first reported as possessing antituberculosis activity, separated from *Streptomyce* sp. G248 obtained from the East Vietnam Sea sponge *Halichondria panicea*. Compounds **198**–**200** had broad-spectrum antibacterial activities, while compounds **201** and **202** were able to inhibit *Mycobacterium tuberculosis*, with MIC values of 6.0 and 11.0 µg/mL, respectively [86]. In order to explore more compounds with broad-spectrum antimicrobial activities, 6-lavandulyl-7-methoxy-5,2′,4′-trihydroxylflavanone (**203**) and 5′-lavandulyl-4′-methoxy-2,4,2′,6′-tetrahydro-xylchalcone (**204**) were firstly isolated from *Streptomyces* sp. G246, making the antimicrobial compounds of actinomycete origin more abundant. Compound **203** significantly inhibited *Escherichia coli*, *Pseudomonas aeruginosa*, *Salmonella enterica*, *Enterococcus faecalis*, *Staphylococcus aureus*, *Bacillus cereus* and *Candida albicans*, with MIC values of 16-128 µg/mL, and compound **204** inhibited all bacteria except *E. coli*, with MIC values of 1-32 µg/mL [87].

**Figure 23:**
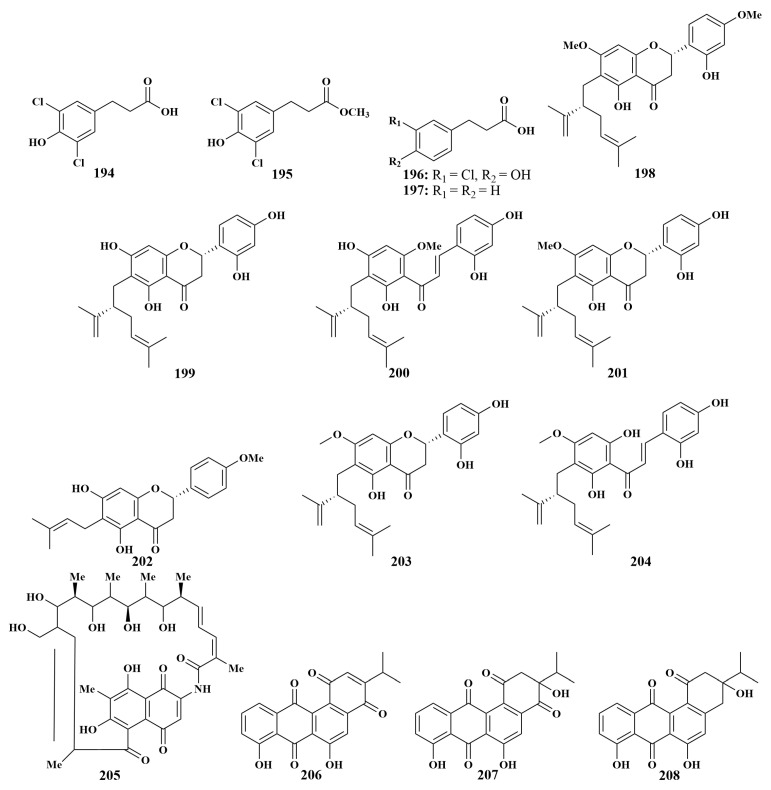
Structures of **194**–**208**.

Satyendra Singh et al. reported the isolation of a known compound rifamycin W (**205**) in 2014 from *Salinispora* sp. FS-0034 obtained from the sponge *Theonella* sp. (Fiji Islands). This compound showed extensive antibacterial activities against MRSA, wild-type *Staphylococcus aureus* and vancomycin-resistant *Enterococcus faecium*, with MIC values of 15.62, 7.80 and 250.00 µg/mL, respectively [88]. *Nocardiopsis* sp. HBJ378, derived from the same genus sponge as **205**, led to the identification of three new angucyclines, nocardiopsistin A–C (**206**–**208**) (Figure 23). All compounds had antibacterial activity against MRSA, with MIC values ranging from 3.12 to 12.5 µg/mL [89].

#### 2.2.2. Alkaloids

In order to find more novel alkaloids in the East China Sea sponge-associated actinomycetes, two same-genus actinomycete strains, identified as *Verrucosispora* sp. FIM06025 and *Verrucosispora* sp. FIM06-0036, produced (2-(hydroxymethyl)-3-(2-(hydroxymethyl)-3-me-thylaziridin-1-yl) (2-hydroxyphenyl) methanone (**209**) and 2-ethylhexyl 1H-imidaz- ole-4-carboxylate (**210**), respectively. Compound **209** exhibited a broad spectrum of antimicrobial activities, with MIC values ranging from 3.4 to 200 µg/mL against *Helicobacter pylori*, *Pseudomonas aeroginosa*, *Acinetobacter baumanniiin*, *Escherichia coli*, *Klebsiella pneumonia*, *Staphylococcus aureus*, *Candida albicans* and *Enterococcus faecium*. Compound **210** exerted antimicrobial activities against *H. pylori*, *K. pneumonia*, *S. aureus* and *E. faecium*, with MIC values of 8, 64, 16 and 256 µg/mL, respectively [90,91].

**Figure 24:**
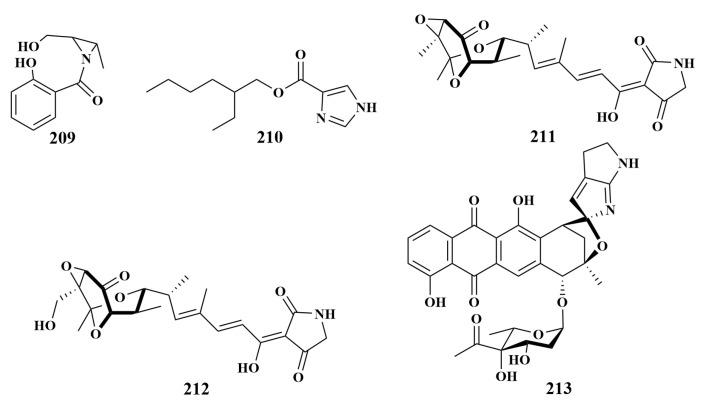
Structures of **209**–**213**.

Tirandamycins A (**211**) and B (**212**) were purified from the culture broth of *Streptomyces tirandamycinicus* sp. nov. HNM0039T, which displayed potent inhibitory activity against *Streptococcus agalactiae*, and the MIC values were 2.52 and 2.55 µg/mL. They also showed antibacterial activity against *Bacillus subtilis*, with MIC values of 5.5 and 6.8 µg/mL, while they were inactive against *Staphylococcus aureus* and *Escherichia coli* at 128 µg/mL [92]. A known compound isoquinocycline B (**213**) (Figure 24) from *Micromonospora ferruginea* sp. nov. 28ISP2-46T, is regarded as a novel source of microorganisms associated with the deep sea sponge. In addition, it possesses better antibiotic activities against *S. aureus*, *Klebsiella pneumoniae* and *Acinetobacter baumannii*, with MIC values of 1.56, 12.50 and 25.00 µM [93].

An examination of the *Rhodococcus* sp. UA13, which was obtained from the Red Sea sponge *Callyspongia aff. Implexa*, revealed a new azepino-di-indole alkaloid, rhodozepinone (**214**), and exhibited significant in vitro antibacterial activity against *Staphylococcus aureus*, with an IC_50_ value of 8.9 µg/mL [94]. A new diketopiperazine, actinozine A (**215**), along with three known compounds cyclo(2-OH-_D_-Pro-_L_-Leu) (**216**), cyclo(_D_-Pro-_L_-Phe) (**217**) and cyclo(_L_-Pro-_L_-Phe) (**218**), were purified from *Streptomyces*. Call-36 also associated with the Red Sea sponge *Callyspongia* species. Compounds **215** and **216** were strongly active against *S. aureus*, with inhibition zones of 23.0 and 20.0 mm. Additionally, these compounds also displayed obvious activity against *Candida albicans*, with inhibition zones of 19.0 and 16.0 mm. Otherwise, compounds **217** and **218** displayed moderate activities against *S. aureus* and *C. albicans*, with inhibition zones of 9.0 to 14.0 mM, respectively [95]. To further explore more diverse diketopiperazines, cyclo(_L_-Phe-*trans*-4-OH-_L_-Pro) (**219**) and cyclo(_L_-Phe-cis-4-OH-_D_-Pro) (**220**) were discovered from the same actinomycete as **194**–**197**, which can also inhibit *S. aureus*, *Escherichia coli* and *C. albicans* [85].

Except for **191**, *Streptomyces* sp. LS298 also led to another two important compounds, maculosin (**221**) and diketopiperazines (**222**), which also act against *Bacillus subtilis*, with inhibition circle diameters of 12 and 13 mM, respectively [83]. Alkhalifah D et al. reported the isolation of two known compounds, phencomycin (**223**) and tubermycin B (**224**), in 2020 from *Streptomyces* sp. RM66 obtained from the sponge *Amphimedon* sp. (Egypt). Phencomycin (**223**) had inhibitory activities against *Bacillus subtilis* and *Escherichia coli*, while tubermycin B (**224**) had inhibitory activity against *Candida albicans* [96].

**Figure 25:**
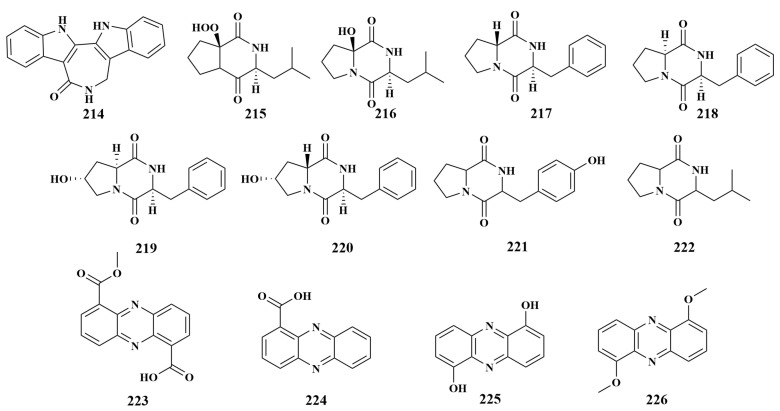
Structures of **214**–**226**.

Two new phenazines, 1,6-dihydroxy phenazine (**225**) and 1,6-dimethoxy phenazine (**226**) (Figure 25), were discovered from *Nocardiopsis* sp. 13-33-15 associated with an unidentified sponge obtained from the South China Sea. Compounds **225** and **226** effectively inhibited the growth of *Bacillus mycoides*, *Staphylococcus aureus*, *Escherichia coli* and *Micrococcus luteus*, with inhibition zones ranging from 8 to 25 mM [97].

#### 2.2.3. Peptides

Besides **191**, **221** and **222**, *Streptomyces* sp. LS298 also produced echinomycin (**227**). Furthermore, obvious activity of 21 mM against *Bacillus subtilis* was observed for compound **227** [83]. In the continuing search for novel biologically active secondary metabolites in this strain, a new analogue of echinomycin containing bicyclic peptide Quinomycin G (**228**) was discovered. It displayed mild antibacterial activities against *Staphylococcuse pidermidis*, *Staphylococcus aureus*, *Enterococcus faecium* and *Enterococcus faecalis*, with MIC values ranging from 16 to 64 μg/mL [98].

**Figure 26:**
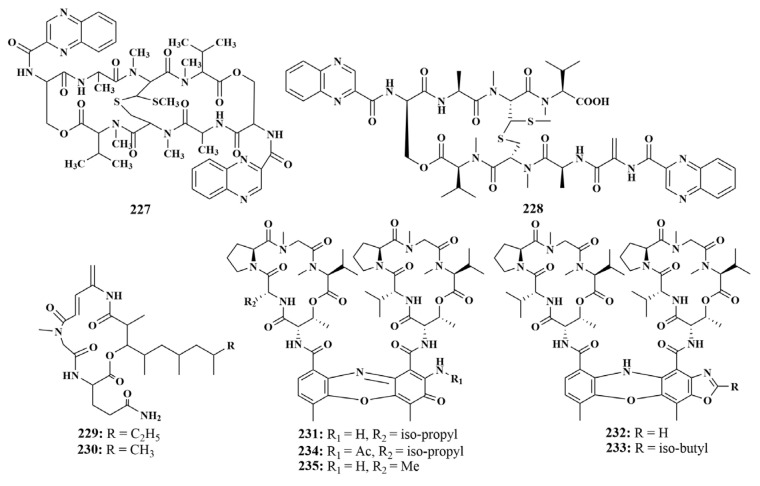
Structures of compounds **227**–**235**.

In a further chemical investigation of the *Streptomyces* sp. actinomycetes, a new cyclic depsipeptide, rakicidin F (**229**), and a known compound, rakicidin C (**230**), were isolated from *Streptomyces* sp. GKU 220 obtained from Thailand sponge. Among them, rakicidin F (**229**) showed growth-inhibitory activities against *Bacillus subtilis* and *Escherichia coli* at a dosage of 25 μg per disk, and rakicidin C (**230**) showed weak activity only against *B. subtilis*, with an MIC value of 50 μg per disk [99]. The actinomycin D (**231**), along with four new D-type actinomycin analogues, actinomycin D_1_–D_4_ (**232**–**235**) (Figure 26), are associated with the sponge *Phyllospongia foliascens* (Xisha islands, China). All compounds were reported to have antimycobacterial activity against MRSA, with MIC values ranging from 0.125 to 1.0 µg/mL [100].

**Figure 27:**
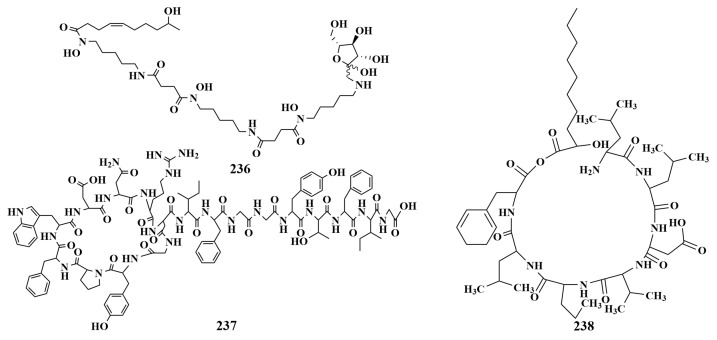
Structures of **236**–**238**.

A new analogue of deferoxamine (**236**) with additional acyl and sugar moiety was separated from *Streptomyces albus* PVA94-07 (Trondheim fjord), which showed a 52% to 56% inhibition of *Escherichia coli* at 16 µg/mL [101]. In 2016, Norimasa Takasaka et al. reported the isolation and identification of a new peptide named actinokineosin (**237**) from *Actinokineospora spheciospongiae* DSM45935T (Germany), which showed antibiotic activity against *Micrococcus luteus* at a dosage of 50 μg per disk [102]. Nesfactin (**238**) (Figure 27) was separated from *Nesterenkonia* sp. MSA31 and was found to show significant antibacterial activity against *Pseudomonas aeruginosa* [103].

#### 2.2.4. Other Nitrogen-Containing Metabolites

**Figure 28:**
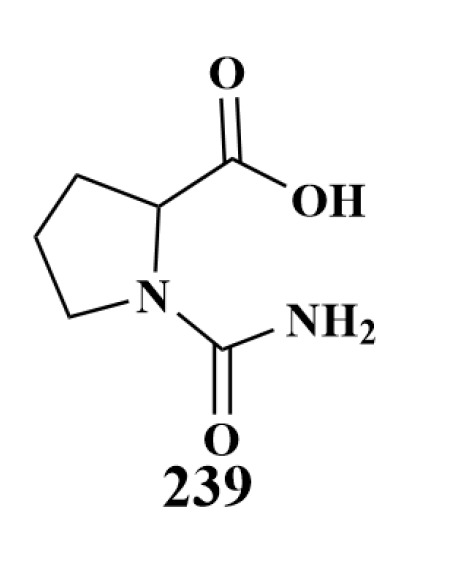
Structure of **239**.

D. N. Naik et al. reported the isolation of a known compound, cinnamic acid (**239**), (Figure 28) in 2013 from *Streptomyces* sp. NIO10068 associated with an unidentified sponge (India), which displayed extensive antibacterial activity against *Pseudomonas aeruginosa* [104].

#### 2.2.5. Glucosides

Urdamycin E (**240**) was also separated from *Streptomycetes* sp. BCC45596 as **186**–**188**, and it strongly inhibited *Mycobacterium tuberculosis* [79]. A novel compound, kocurin (**241**), was found by *Kocuria palustris* F276,345, which was associated with an unidentified sponge (Florida Islands). Kocurin (**241**) had extremely potent activity against MRSA, with MIC values of 0.25 to 0.5 μg/mL [105]. Microluside A (**242**), which was extracted from *Micrococcus* sp. EG45 associated with the sponge *Spheciospongia vagabunda* collected from the Red Sea, showed antibacterial potential against *Enterococcus faecalis* and *Staphylococcus aureus*, with MIC values of 10 and 13 μM, respectively [106].

**Figure 29:**
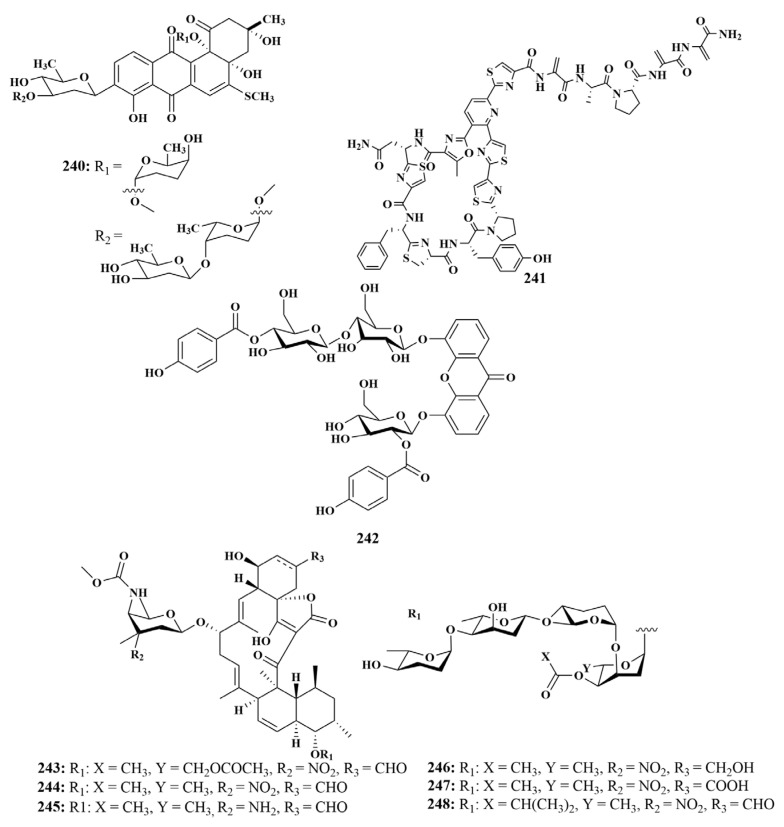
Structures of **240**–**248**.

In 2018, Gong Ting et al. reported the isolation and identification of a new spirotetronate glycoside, tetrocarcin Q (**243**), along with six known analogues tetrocarcin A (**244**), AC6H (**245**), tetrocarcin N (**246**), tetrocarcin H (**247**) and arisostatin A (**248**) (Figure 29) from *Micromonospora carbonacea* LS276 associated with the sponge *Gelliodes carnosa* (Hainan, China). All compounds exhibited antimicrobial activity against *Bacillus subtilis*, with MIC values ranging from less than 0.048 to 50 µM, with **207** and **211** showing strong antibacterial activity. In this article, it was found for the first time that compound **243** had a unique oligosaccharide chain at the C-9 position [107].

### 2.3. Marine Natural Products with Antimicrobial Activity from Sponge-Associated Bacterium

Marine bacteria are the most widely distributed and abundant group of microorganisms in the ocean. However, previous studies found that few species of associated bacteria (except for the actinomyces) have been isolated from sponges, and most of the current studies have focused on sponge-associated fungi and sponge-associated actinomycetes. Therefore, the discovery of antimicrobial active secondary metabolites from sponge-associated bacteria (except for the actinomyces) has a broader developmental prospect.

#### 2.3.1. Polyketides

**Figure 30:**
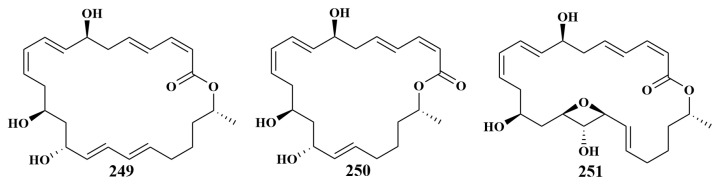
Structures of **249**–**251**.

Dat. T. T. H reported the isolation of three new compounds, macrolactin A (**249**), macrolactin H (**250**) and 15,17-epoxy-16-hydroxy macrolactin A (**251**) (Figure 30), in 2021 from *Bacillus* sp. M1_CRV_171 obtained from Vietnamese sponge. Compound **249** exhibited antimicrobial activities against *Pseudomonas aeruginosa*, *Staphylococcus aureus* and *Rhodococcus* sp., with MIC values of 8, 16 and 32 µg/mL, respectively. Compound **250** showed antimicrobial activities against *E. coli* and *S. aureus*, with MIC values of 16 and 32 µg/mL, respectively. For compound **251**, the strongest activity was found against *E. coli*, with an MIC value of 32 µg/mL [108].

#### 2.3.2. Alkaloids

**Figure 31:**
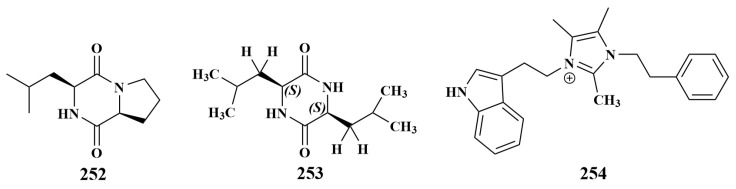
Structures of **252**–**254**.

The isolation of cyclo-(l-Leu-l-Pro) (**252**) was reported in 2015 from *Pseudomonas fluorescens* associated with the sponge *Isodictya setifera*, which displayed antibacterial activities against *Staphylococcus aureus* and *Pseudomonas aeruginosa*, with equal MIC values of 512 μg/mL [109]. A new diketopiperazine, (3S,6S)-3,6-diisobutylpiperazine-2,5-dione (**253**), was separated from *Bacillus* sp. SPB7 associated with the sponge *Spongia officinalis*, which showed strong antimicrobial activities against *Escherichia coli* and *S. aureus*, with MIC values of 16 and 22 µg/mL, respectively. This compound was the first isolated from sponge-associated bacteria and also constitutes the first report of its antibacterial activity, which provides a new perspective for natural product drug development [110]. *Bacillus* sp. WMMC-1349 was recovered from *Cinachyrella apion*, from which bacillimidazole F (**254**) was discovered, which exhibited weak antibiotic activity against MRSA, with an MIC of 38.3 µM. This report was the first to identify **254** (Figure 31) as a natural product containing an imidazole heterocycle [111].

#### 2.3.3. Peptides

**Figure 32:**
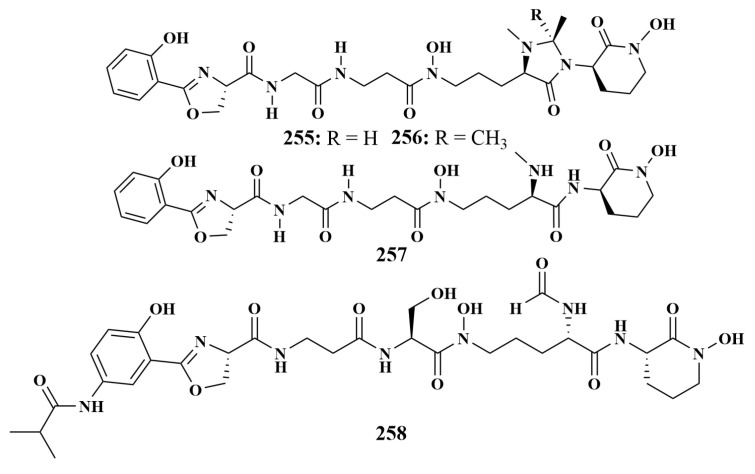
Structures of **255**–**258**.

Two new siderophores, madurastatins D1 (**255**) and D2 (**256**), together with a known compound (−)-madurastatin C1 (**257**) containing the additional heterocyclic structure from *Actinomadura* sp. WMMA-1423, are associated with the sponge *Tedania* sp., which displayed moderately antibacterial activity against *Micrococcus garciniae* [112]. A new clamp iron compound, pseudonochelin (**258**) (Figure 32), was reported in 2022 from *Pseudonocardia* sp. WMMC-193 associated with the sponge *Lissodendoryx stigmata* (Florida), and can inhibit MRSA and methicillin-sensitive *Staphycoccus aureus* (MSSA), with MIC values of 4 µg/mL, respectively [113].

### 2.4. Marine Natural Products with Antimicrobial Activity from Sponge-Associated Strains through Co-Cultivation Method

With the increasing duplication of secondary metabolites produced by traditional fermentation methods, many researchers have started to search for new fermentation methods, and co-cultivation is one of them. By isolating an associated strain from the sponge and putting it in a culture with another strain, silent genes are activated and new secondary metabolites are produced.

#### 2.4.1. Polyketides

In 2019, the associated fungus *Aspergillus versicolor* was isolated from the sponge *Agelas oroides* collected from Turkey and co-cultivated with *Bacillus subtilis* to obtain four known compounds, versicolorin B (**259**), diorcinol D (**260**), diorcinol G (**261**) and diorcinol I (**262**). These compounds were able to selectively inhibit *Staphylococcus aureus*, *Enterococcus faecalis*, *Enterococcus faecium*, *Fusarium putrefaciens*, *Bacillus subtilis* and *Escherichia coli*, with MIC values of 6.25 to 100 µg/mL. This article gave an insight into the ability to target the activation of fungal silencing genes using the co-cultivation strategy [114].

**Figure 33:**
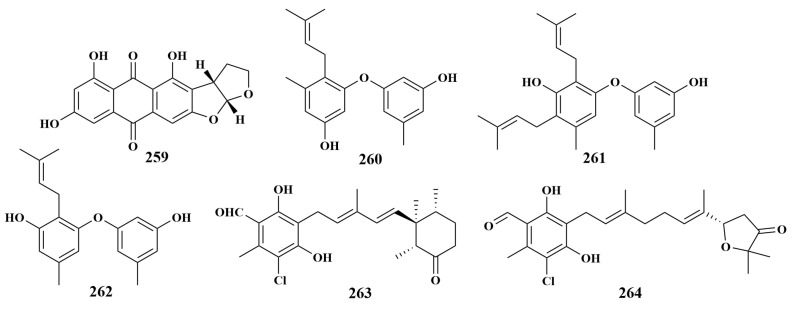
Structures of **259**–**264**.

In 2021, the sponge-associated fungus *Acremonium* sp. IMB18086 and *Pseudomonas aeruginosa* were co-cultivated to obtain two known compounds, ascochlorin (**263**) and ascofuranone (**264**) (Figure 33), which showed significant antibacterial activities against *Staphylococcus aureus*, *Bacillus subtilis*, MRSA and *Candida albicans* [115].

#### 2.4.2. Alkaloids

In 2014, Dashti et al. cultured the Mediterranean sponge *Dysidea avara*-associated actinomycete *Nocardiopsis* sp. RV163 with the Red Sea sponge *Spheciospongia vagabunda*-associated actinomycete *Actinokineospora* sp. EG49 to produce a new diketopiperazine, 1,6-dihydroxyphenazine (**265**), which exhibited antibiotic activity against *Bacillus subtilis*, with an inhibition circle diameter of 11 mm. This was the first report on the induction of secondary metabolites by the co-cultivation of two sponge-associated actinomycetes, which was an important guideline for the study of new fermentation strategies [116].

**Figure 34:**
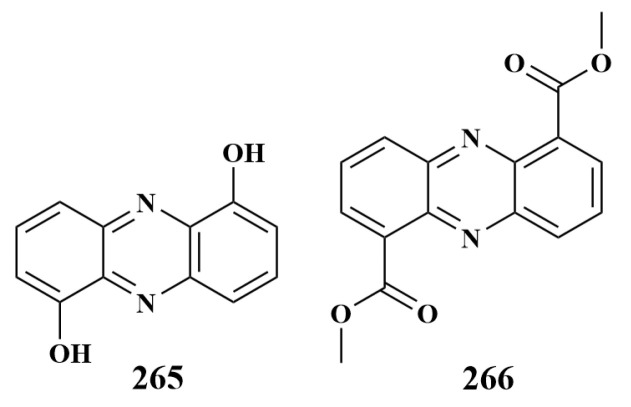
Structures of **265**–**266**.

In 2020, Li et al. isolated two Red Sea sponges, *Callyspongia* sp. and *Spheciosponge vagabunda*, which were isolated from the associated actinomycetes *Micromonospora* sp. UR56 and *Actinokineospora* sp. EG49, respectively. They were isolated by culturing them together to obtain the compound dimethyl phenazine-1,6-dicarboxylate (**266**) (Figure 34), which was able to significantly inhibit *Staphylococcus aureus*, *Bacillus subtilis*, *Pseudomonas aeruginosa* and *Escherichia coli*, with 47% to 94% inhibition rates [117].

#### 2.4.3. Peptides

**Figure 35:**
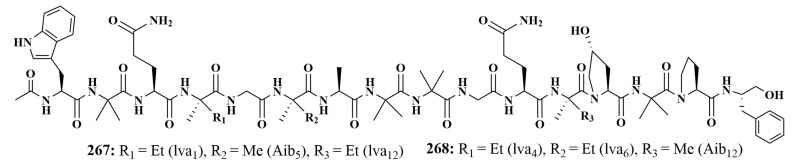
Structures of **267**–**268**.

The sponge-associated fungi *Acremonium* sp. IMB18086 and *Pseudomonas aeruginosa* were co-cultured, and were also the source of two new pipemycin acremopeptaibols A (**267**) and acremopeptaibols E (**268**) (Figure 35). In addition, they also exhibited remarkable antibacterial activities against *Staphylococcus aureus*, *Bacillus subtilis*, MRSA and *Candida albicans* [115].

#### 2.4.4. Other Nitrogen-Containing Metabolites

**Figure 36:**
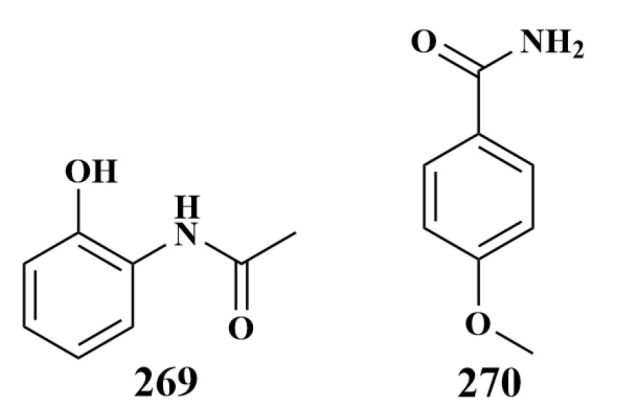
Structures of **269**–**270**.

Except for dimethyl phenazine-1,6-dicarboxylate (**266**), N-(2-hydroxyphenyl)-acetamde (**269**) and p-anisamide (**270**) (Figure 36) were also isolated from these two Red Sea sponges and had the same antimicrobial activities as **266**. The bioassay test indicated that the presence of carboxylic acid or ester groups at C-1 and C-6 of these compounds was shown to be essential for the antibacterial effects [117].

## 3. Discussion

**Figure 37:**
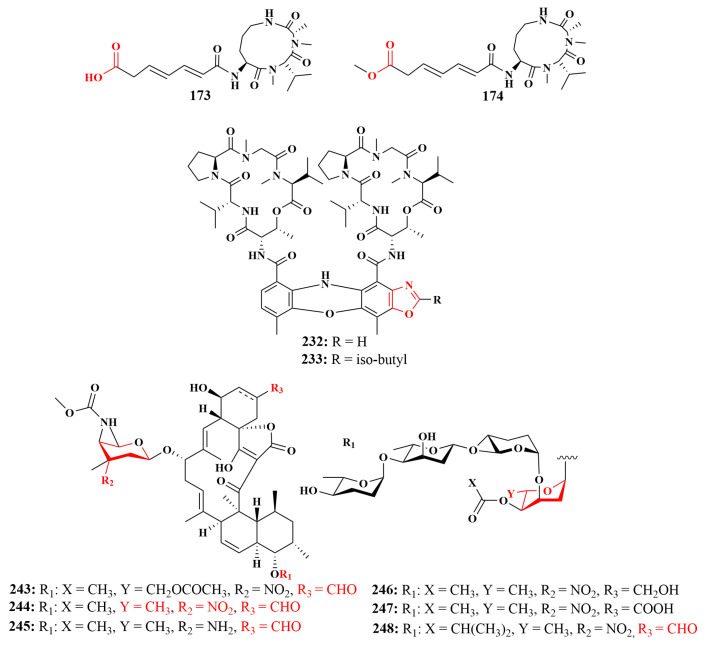
Effect of the structure-activity relationship of compounds on antimicrobial activity.

In a further investigation of antimicrobial activity, the structural differences of the compounds will have an important impact. For instance, compounds **173** and **174** showed broad antibacterial activity against a panel of pathogenic strains, whereas compounds **171**, **172** and **175** were less active, which indicates that the carboxyl group or its methyl ester have an important role in antibacterial activities [66]. The anti-MRSA activities of **232** and **233** were nearly two to four times more potent than **231**, **234** and **235**, which suggests that the activity might be enhanced by the incorporation of an additional oxazole unit into the phenoxazinone chromophore [89]. Except for the above compounds, compound **243** had a lower antimicrobial activity than **244**, implying that 6-CH_3_ of sugar B in the oligosaccharide chain at C-9 played a key role in the antibacterial activity. Furthermore, compound **245** was at least 10-fold less active than **244**, suggesting that the NO_2_ sugar was important for the antibacterial activity. Compounds **246** and **247** were less active than **244**, **245** and **248**, inferring that the aldehyde group at C-23 was also essential for the activity [96] (Figure 37). Through the structure-activity relationship of compounds, we can provide a theoretical basis for the study of antibacterial mechanisms.

Moreover, some novel compounds possess the potential to be developed as antimicrobial and anticancer agents, such as **116**, **117**, **120**, **163** and **168**. Among them, fonsecinone A (**116**) and isoaurasperone A (**117**) showed the same antibacterial activity as the positive control, ampicillin sodium [58]. Meanwhile, isochaetochromin B_2_ (**40**) and ustilaginoidin D (**41**) also showed the same activity against *M. phlei* as the positive control, streptomycin sulphate [33]. In addition, a few compounds have broad-spectrum antibacterial activity; for example, compounds **28**, **29**, **89**, **90**, **91**, **127**–**136**, **167**, **209**, **228** and **242**, which mainly inhibit *Escherichia coli*, *Staphylococcus aureus*, *Klebsiella pneumoniae*, *Pseudomonas aeruginosa*, *Enterococcus faecalis* and so on. These compounds are expected to replace the original antibiotics in the treatment of diseases caused by MDR strains, and provide good lead compounds for solving the problem of antibiotic resistance.

Remarkably, it was found that the antimicrobial active compounds of sponge-associated microorganisms have a variety of potential targets. The drug-like properties and binding potential of several sponge-microorganisms-derived lead molecules against the VP40 target of Ebola virus were hypothesized by computational virtual screening and molecular docking [10]. In addition, Fernanda et al. used breast cancer cell lines MCF7, SKBR3 and MDA-MB-231 and a non-tumor cell line MCF12A for experiments in vitro, and found that preussin (**166**) showed cytotoxic and antiproliferative activities in breast cancer cell lines in 2D and 3D cultures [118]. Pang et al. performed experiments using nasopharyngeal carcinoma cell line CNE2 and a xenograft murine model, and found that physcion (**89**) suppressed the growth of nasopharyngeal carcinoma cells in vitro and in vivo. Moreover, physcion (**89**) had anti-proliferative effects by modulating Sp1 via the generation of ROS and regulating the miR-27a/ZBTB10 axis [119]. In 2022, Liu et al. found that physcion (**89**) also had a good antibacterial effect against *Chlamydia psittaci*. By conducting in vivo and in vitro experiments with *C. psittaci* infection in avian animals, they found that the animals treated with physcion (**89**) had alleviated dyspnea and lesions of air sacs and lungs, as well as reduced bacterial loads in spleens, which was comparable to doxycycline treatment. By exploring it antibacterial mechanism, physcion (**89**) could block *Chlamydial* adhesion to host cells, RB-to-EB differentiation and the activation of bacterial autophagy. Thus, it will be a good alternative to doxycycline in combating virulent *C. psittaci* infection, contributing to the eradication of *Chlamydial* transmission from animals to human beings [120]. Oliveira et al. found that the combination of citrinin (**42**) and vancomycin could significantly improve the survival rate of VRE-infected mice by inhibiting the activity of the mesoderm, thus providing a new treatment and idea for diseases caused by VRE [121]. We can explore whether the antimicrobial active compounds isolated from sponge-associated microorganisms can be used in combination with other commercial antimicrobials to provide new options for mitigating the resistance of pathogenic bacteria.

In addition, except for traditional analytical methods, more and more new techniques have been used to obtain secondary metabolites of sponge-associated microorganisms, such as the structure and relative configuration of new compounds being determined by means of quantum mechanical nuclear magnetic resonance (QM-NMR) computational assistance technology and the method of the mass spectrometry (MS)/MS data construction of a molecular network [23]. The absolute configuration of the compound was determined by time-dependent density functional theory/electronic circular dichroism (TDDFT-ECD), and the target compound was found using high-throughput sequencing. This opens up the possibility of finding more antibacterial active compounds of sponge-associated microorganisms.

It is of concern that a small number of sponge-microorganisms-derived active compounds have been studied or predicted with their drug likeliness, pharmacokinetics and toxicity in order to evaluate their potential for drug development. For example, Skariyachan et al. predicted the drug likeliness properties of selected lead compounds by the web-based application PreADMET. Then, pharmacokinetics and ADME (adsorption, distribution, metabolism and excretion) the properties of them that qualified as drug likeness properties were predicted, followed by a toxicity prediction [10]. Butyrolactone I (BTL-I),an active compound obtained from sponge-derived *Aspergillus* fungi [122] (also found in fungi from other sources), was studied with its metabolic and pharmacokinetic profile in rats using ultra-high-performance liquid chromatography–quadrupole time-of-flight mass spectrometry (UHPLC-Q-TOF-MS) and UHPLC-MS/MS methods. The results show that the oral bioavailability was calculated as 6.29%, and the maximum plasma concentrations were 9.85 ± 1.54 ng/mL and 17.97 ± 1.36 ng/mL for intravenous and intragastric dosing groups, respectively. The major metabolic pathways were oxidative and glucuronide conjugation [123]. Although BTL-I showed attractive biological activities and an appropriate pharmacokinetic profile, its adverse effects should not be neglected. Our group found that BTL-I could significantly increase the mRNA and protein levels of oncogenes such as CYP1A1, CYP3A7, ALDH3A1 and GSTA2 in HepG2 cells. Thus, the chemical carcinogenesis or toxicity issue may be of particular importance or even fatal in its drug development [124]. 

In summary, the antimicrobial active substances of sponge-associated microorganisms have broad application prospects and great potential to develop into new antibiotics. Therefore, more attention should be paid to the molecular mechanism and further in vivo and preclinical studies, which will provide the possibility of discovering new drugs.

## 4. Conclusions and Perspectives

**Figure 38:**
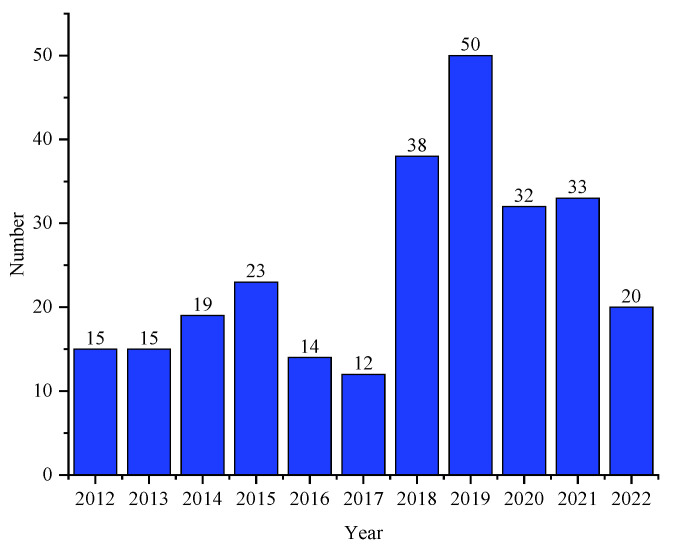
Statistics on the number of secondary metabolites of sponge-associated microorganisms from 2012–2022.

**Figure 39:**
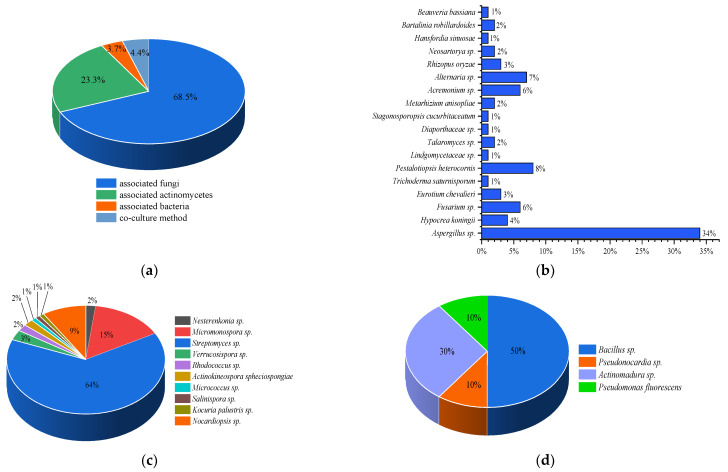
(**a**) Sources of secondary metabolites; (**b**) strains of sponge-associated fungi; (**c**) tsrains of sponge-associated actinomycetes; (**d**) strains of sponge-associated bacteria.

**Figure 40:**
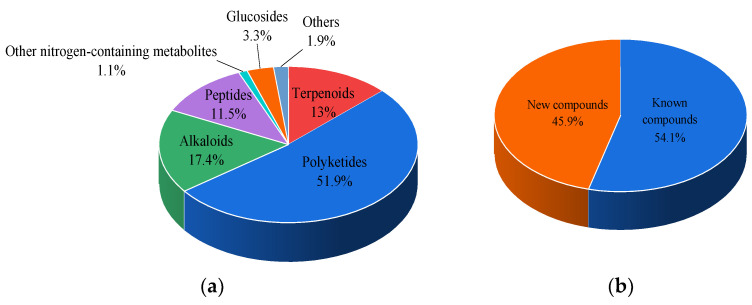
(**a**) Structural diversity distribution; (**b**) statistics of new and known secondary metabolites.

Marine natural products have contributed significantly to modern drug development. We summarized the sources, structural diversity and antimicrobial activity of 270 newly reported secondary metabolites from the sponge-associated microorganisms according to a survey of the literature published from 2012 to 2022 (Figure 38). At the domain level, 68.5% of the natural products were derived from fungi, 23.3% originated from actinomycetes, 3.7% were obtained from other bacteria and 4.4% were discovered using a co-culture method (Figure 39a), of which Aspergillus, Streptomyces and Actinomadura were the main source of natural products (Figure 39b–d). The chemical structures of the derived compounds were divided into seven categories as shown in Figure 40a. Remarkably, there are 124 new compounds and 146 known compounds (Figure 40b), 55 of which have antifungal activity in addition to antipathogenic bacteria.

Furthermore, great progress has been made in the study of secondary metabolites of sponge-associated microorganisms in recent years. However, many studies contain few antibacterial active substances and they have a single source. On the basis of previous studies, we summarized these antimicrobial active compounds and discussed the structure–activity relationship, potential targets, drug likeliness, pharmacokinetics, toxicity potential and features of some compounds for therapeutic purposes. In addition, many compounds also have anti-phytopathogenic fungal activity, which provides a theoretical basis for the research and development of agricultural fungicides. We hope to isolate more strains of sponge-associated microorganisms and discover novel natural products through new culture strategies and techniques, leading to the development of new drugs.

## Figures and Tables

**Table 1:** Sponge origin and antimicrobial activity of all compounds.

Sponge Origin	Compounds	Antimicrobial Activity
*Agelas oroides*	**102**, **103**, **104**	*Enterococcus faecalis* (*E. faecalis*), *Staphylococcus aureus* (*S. aureus*), *Enterococcus faecium* (*E. faecium*)
	**259**, **260**, **261**, **262**	*E. faecalis, S. aureus, E. faecium, Fusarium solani* (*F. solani*), *Escherichia coli* (*E. coli*), *Bacillus subtilis* (*B. subtilis*)
*Amphimedon* sp.	**223**, **224**	*B. subtilis, E. coli, Candida albicans* (*C.albicans*)
*Axinella* sp.	**168**	*Staphylococcus epidermidis* (*S. epidermidis*)
	**48**, **49**, **50**	*S. epidermidis,* methicillin-resistant *Staphylococcus aureus* (MRSA)
*Callyspongia* sp.	**122**, **123**, **124**	*S. aureus, Vibrio parahaemolyticus* (*V. parahaemolyticus*)
	**125**, **214**	*S. aureus*
	**30**, **31**, **183**	*S. aureus, Acinetobacter baumannii* (*A. baumannii*)
	**126**, **146**, **147**	*S. aureus,* MRSA
	**127-136**, **184**, **185**	*E. coli, Vibrio proteolyticus* (*V. proteolyticus*), *Proteus mirabilis* (*P. mirabilis*), *Pseudomonas fluorescens* (*P. fluorescens*), *Shigella flexneri* (*S. flexneri*), *Salmonella cholerasuis* (*S. cholerasuis*), *Listeria monocytogenes* (*L. monocytogenes*), *S. aureus*
	**215**, **216**, **217**, **218**	*S. aureus, C. albicans*
	**194**–**197**, **219**, **220**	*S. aureus, E. coli, C. albicans*
*Cinachyrella apion*	**254**	MRSA
*Ciocalypta* sp.	**17**, **18**, **19**	*Vibrio scophthalmi* (*V. scophthalmi*), *Vibrio shilonii* (*V. shilonii*), *Vibrio brasiliensis* (*V. brasiliensis*)
*Clathria reinwardtii*	**9**, **143**	*S. aureus,* vancomycin-resistant *Enterococcus* (VRE) *E. faecalis*, *E. faecalis*
*Dictyonella incisa*	**22**, **23**	VRE, *B. subtilis*
*Dendrectilla tremitersis*	**105**–**109**	*S. aureus,* MRSA, *E. coli, P. aeruginosa*
*Dysidea* sp.	**137**, **138**	*S. aureus,* MRSA, *Cryptoccus neoformans* (*C. neoformans*), *Microsporum gypseum* (*M. gypseum*)
	**190**	*Chlamydia trachamatis* (*C. trachamatis*)
*Epipolasis* sp.	**163**	*S. aureus, E. faecalis,* MRSA, VRE *E. faecium*
*Gelliodes carnosa*	**76**, **77**, **78**	*S. epidermidis, S. aureus, B. subtilis*
	**191**, **221**, **222**, **227**, **243**–**248**	*B. subtilis*
	**228**	*S. epidermidis, S. aureus, E. faecium, E. faecalis*
*Grantia compressa*	**28**, **29**, **89**, **90**, **101**, **167**	*S. aureus, E. faecalis, E. coli, P. aeruginosa, Bacillus cereus* (*B. cereus*), *Micrococcus luteus* (*M. luteus*), *Salmonella typhimurium* (*S. typhimurium*), *Mycobacterium tuberculosis* (*M. tuberculosis*), *Klebsiella pneumoniae* (*K. pneumoniae*), *Streptococcus pneumoniae* (*S. pneumoniae*)
*Halichondria panicea*	**198**–**202**	*E. faecalis, S. aureus, E. coli, B. cereus, P. aeruginosa, Salmonella enterica* (*S. enterica*), *C. albicans, M. tuberculosis*
	**203**, **204**	*E. faecalis, S. aureus, E. coli, B. cereus, P. aeruginosa, S. enterica, C. albicans*
*Haliclona* sp.	**25**, **26**, **27**	*Vibrio harvey* (*V. harvey*)
	**110**, **111**	*S. aureus, E. coli*
	**118**, **119**, **120**, **121**	*S. aureus, E. coli, B. subtilis,* MRSA, *M. tuberculosis*
	**112**, **113**	*C. albicans, C. neoformans*
	**114**	*M. tuberculosis, S. aureus, E. coli, B. subtilis*
	**164**, **165**	*B. subtilis*
*Hymeniacidon perleve*	**82**	*S. aureus, B. subtilis*
	**83**, **84**	MRSA, *S. aureus, B. subtilis,* Bacille Calmette Guerin (BCG)
*Hyrtios erectus*	**81**, **156**, **157**, **158**	*S. aureus, E. coli, C. albicans*
*Isodictya setifera*	**252**	*S. aureus, P. aeruginosa*
*Lissodendoryx stigmata*	**258**	MRSA, methicillin-sensitive *Staphycoccus aureus* (MSSA)
*Melophlus* sp.	**42**	MRSA, *S. aureus,* VRE *E. faecium, C. neoformans*
*Mycale* sp.	**95**–**100**	*S. aureus, E. faecalis,* VRE *E. faecalis,* MRSA
	**101**	*E. faecalis*
*Neopetrosia* sp.	**93**, **94**	VRE *E. faecalis, S. aureus, E. faecalis,* MRSA
*Niphates* sp.	**51**, **52**	*Pseudomonas lachrymans* (*P. lachrymans*), *Agrobacterium tumefaciens* (*A. tumefaciens*), *Xanthomonas vesicatoria* (*X. vesicatoria*), *Ralstonia solanacearum* (*R. solanacearum*), *S. aureus*
	**32**, **33**, **34**, **35**	*S. aureus*
*Phakellia fusca*	**24**, **180**, **181**	*S. aureus*
	**53**, **54**, **55**, **56**, **57**, **58a/b**	*S. aureus, B. subtilis, C. albicans, C. neoformans, Candida parapsilosis* (*C. parapsilosis*)
	**59**–**67**	*S. aureus, B. subtilis, C. neoformans, C. parapsilosis*
	**72**, **73**, **74**, **75**	*S. aureus,* MRSA, *E. coli*
	**176**, **177**, **178**, **179**	*Aspergillus fumigatus* (*A. fumigatus*), *Aspergillus niger* (*A. niger*)
*Phomopsis* sp.	**10**	*S. aureus,* MRSA
*Phyllospongia foliascens*	**231**–**235**	MRSA
*Reniera japonica*	**116**, **117**	*Helicobacter pylori* (*H. pylori*)
*Rhabdermia* sp.	**8**	MRSA
*Scopalina ruetzleri*	**189**	*B. subtilis, Mycolicibacterium smegmatis* (*M. smegmatis*)
*Spheciosponge vagabunda*	**242**	*E. faecalis, S. aureus*
	**265**	*B. subtilis*
	**266**, **269**, **270**	*S. aureus, B. subtilis, P. aeruginosa, E. coli*
*Spongia officinalis*	**253**	*E. coli, S. aureus*
*Stelletta* sp.	**139**, **140**, **141**, **142**	*S. aureus, Streptococcus iniae* (*S. iniae*), *Vibrio ichthyoenteri* (*V. ichthyoenteri*)
*Suberea* sp.	**79**, **80**	*S. aureus, B. subtilis, Bacillus megaterium* (*B. megaterium*), *M. smegmatis*
	**148**, **149**	*C. albicans, S. aureus, E. coli*
*Tedania* sp.	**255**, **256**, **257**	*M. luteus*
*Tethya aurantium*	**45**, **46**, **47**	*C. albicans, Septoria tritici* (*S. tritici*), *Trichophyton rubrum* (*T. rubrum*), *B. subtilis, Staphylococcus lentus* (*S. lentus*)*,* MRSA
	**11**, **12**, **13**, **92**, **159**, **160**, **161**	*S. aureus, Halomonas aquamarina* (*H. aquamarina*), *Polaribacter irgensii* (*P. irgensii*), *Pseudoalteromonas elyakovii* (*P. elyakovii*), *Roseobacter litoralis* (*R. litoralis*), *Shewanella putrefaciens* (*S. putrefaciens*), *Vibrio natriegens* (*V. natriegens*), *V. harvey, Vibrio carchariae* (*V. carchariae*), *V. proteolyticus*
*Theonella* sp.	**205**	MRSA, wild-type *Staphylococcus aureus* (WTSA), VRE *E. faecium*
	**206**, **207**, **208**	MRSA
*Xestospongia* sp.	**1, 2, 3, 4, 5**	*S. aureus, B. subtilis, E. coli, B. cereus, Sarcina lutea* (*S. lutea*), *Micrococcus tetragenus* (*M. tetragenus*), *Vibrio anguillarum* (*V. anguillarum*), *V.parahaemolyticus*
	**6**, **7**, **68**–**71**	*S. enterica*
	**186**, **187**, **188**, **240**	*M. tuberculosis*
Unidentified	**36**, **37**	*C. albicans*
	**38**, **39**, **40**, **41**	*Mycobacterium phlei* (*M. phlei*), *M. tuberculosis*
	**43**, **44**	*B. cereus, V. parahaemolyticus, Streptomyces albus* (*S. albus*)
	**144**, **145**	*C. albicans, C. neoformans, Candida glabrata* (*C. glabrata*)
	**150**, **151**, **152**, **153**	*V. harvey*
	**20**, **21**	*K. pneumoniae, E. faecalis, A. hydrophila*
	**85**, **86**	*B. subtilis, S. aureus,* MRSA, *C. albicans, Propionibacterium acnes* (*P. acnes*), *S. epidermidis, Xanthomonas campestris* (*X. campestris*), *Alternaria alternata* (*A. alternata*)
	**169**, **170**	*Alternaria brassicae* (*A. brassicae*)
	**154**, **155**	*S. aureus, Aeromonas hydrophila* (*A. hydrophila*), *V. harvey, Gaeumannomyces gramini* (*G. gramini*), *V. parahaemolyticus*
	**115**	*S. aureus, M. tuberculosis*
	**14**, **15**, **16**	*Streptococcus agalactiae* (*S. agalactiae*)
	**166**	*V. parahaemolyticus*
	**87**, **88**	*P. aeruginosa, M. phlei,* meticillin-resistant coagulase negative *Staphylococcus* (MRCNS), *B. subtilis, B. cereus, V. parahemolyticus*
	**171**, **172**, **173**, **174**, **175**	*B. cereus, Proteus* sp., *M. phlei*, *B. subtilis*, MRSA, MRCNS, *V. Parahaemolyticus*, *Edwardsiella tarda* (*E. tarda*)
	**162**	*S. epidermidis*
	**238**, **239**	*P. aeruginosa*
	**241**	MRSA
	**225**, **226**	*M. luteus, Bacillus mycoides* (*B. mycoides*), *S. aureus, E. coli*
	**237**	*M. luteus*
	**236**	*E. coli*
	**229**, **230**	*B. subtilis, E. coli*
	**209**	*H. pylori, P. aeroginosa, A. baumanniiin, E. coli, K. pneumonia*, *S. aureus, C. albicans, E. faecium*
	**210**	*H. pylori, K. pneumonia, S. aureus, E. faecium*
	**211**, **212**	*S. agalactiae, B. subtilis, E. coli, S. aureus*
	**192**, **193**	MRSA, *E. coli*
	**213**	*K. pneumonia, S. aureus, A. baumannii*
	**249**, **250**, **251**	*P. aeruginosa, S. aureus, Rhodococcus baikonurensis* (*R. baikonurensis*), *E. coli*
	**267, 268, 263, 264**	*B. subtilis, S. aureus*, MRSA, *C. albicans*

## Data Availability

Not applicable.

## Abbreviations

*S. aureus*	*Staphylococcus aureus*	*E.faecalis*	*Enterococcus faecalis*
*E. tarda*	*Edwardsiella tarda*	*E.faecium*	*Enterococcus faecium*
*F. solani*	*Fusarium solani*	*E.coli*	*Escherichia coli*
*B. subtilis*	*Bacillus subtilis*	*C.albicans*	*Candida albicans*
*S. epidermidis*	*Staphylococcus epidermidis*	MRSA	methicillin-resistant *Staphylococcus aureus*
*V. parahaemolyticus*	*Vibrio parahaemolyticus*	*A.baumannii*	*Acinetobacter baumannii*
*V. proteolyticus*	*Vibrio proteolyticus*	*P.mirabilis*	*Proteus mirabilis*
*P. fluorescens*	*Pseudomonas fluorescens*	*S.flexneri*	*Shigella flexneri*
*S. cholerasuis*	*Salmonella cholerasuis*	*L.monocytogenes*	*Listeria monocytogenes*
*V. scophthalmi*	*Vibrio scophthalmi*	*V.shilonii*	*Vibrio shilonii*
*V. brasiliensis*	*Vibrio brasiliensis*	VRE	vancomycin-resistant *Enterococcus*
*C. neoformans*	*Cryptoccus neoformans*	*M.gypseum*	*Microsporum gypseum*
*C. trachamatis*	*Chlamydia trachamatis*	*B.cereus*	*Bacillus cereus*
*M. luteus*	*Micrococcus luteus*	*S.typhimurium*	*Salmonella typhimurium*
*M. tuberculosis*	*Mycobacterium tuberculosis*	*K.pneumoniae*	*Klebsiella pneumoniae*
*S. pneumoniae*	*Streptococcus pneumoniae*	*S.enterica*	*Salmonella enterica*
*V. harvey*	*Vibrio harvey*	BCG	*Bacille Calmette Guerin*
*P. lachrymans*	*Pseudomonas lachrymans*	MSSA	*methicillin-sensitive Staphycoccus aureus*
*A. tumefaciens*	*Agrobacterium tumefaciens*	*X.vesicatoria*	*Xanthomonas vesicatoria*
*R. solanacearum*	*Ralstonia solanacearum*	*C.parapsilosis*	*Candida parapsilosis*
*A. fumigatus*	*Aspergillus fumigatus*	*A.niger*	*Aspergillus niger*
*H. pylori*	*Helicobacter pylori*	*M.smegmatis*	*Mycolicibacterium smegmatis*
*S. iniae*	*Streptococcus iniae*	*V.ichthyoenteri*	*Vibrio ichthyoenteri*
*B. megaterium*	*Bacillus megaterium*	*S.tritici*	*Septoria tritici*
*T. rubrum*	*Trichophyton rubrum*	*S.lentus*	*Staphylococcus lentus*
*H. aquamarina*	*Halomonas aquamarina*	*P.irgensii*	*Polaribacter irgensii*
*P. elyakovii*	*Pseudoalteromonas elyakovii*	*R.litoralis*	*Roseobacter litoralis*
*S. putrefaciens*	*Shewanella putrefaciens*	*V.natriegens*	*Vibrio natriegens*
*V. carchariae*	*Vibrio carchariae*	WTSA	*wild-type Staphylococcus aureus*
*S. lutea*	*Sarcina lutea*	*M.tetragenus*	*Micrococcus tetragenus*
*V. anguillarum*	*Vibrio anguillarum*	*M.phlei*	*Mycobacterium phlei*
*S. albus*	*Streptomyces albus*	*C.glabrata*	*Candida glabrata*
*P. acnes*	*Propionibacterium acnes*	*X.campestris*	*Xanthomonas campestris*
*A. alternata*	*Alternaria alternata*	*A.hydrophila*	*Aeromonas hydrophila*
*G. gramini*	*Gaeumannomyces gramini*	*S.agalactiae*	*Streptococcus agalactiae*
OSMAC	One Strain Many Compounds	MRCNS	meticillin-resistant coagulase-negative *Staphylococcus*
*B. mycoides*	*Bacillus mycoides*	*R.baikonurensis*	*Rhodococcus baikonurensis*
IC_50_	half-inhibitory concentration	MIC	minimum inhibitory concentration
QM-NMR	quantum mechanical nuclear magnetic resonance	TDDFT	time-dependent density functional theory
MS	mass spectrometry	ECD	electronic circular dichroism
BTL-I	Butyrolactone I	MptpB	*Mycobacterium tuberculosis* protein tyrosine phosphatase B
KEGG	Kyoto Encylopaedia of Genes and Genomes	UHPLC-Q-TOF-MS	ultra-high-performance liquid chromatographyqua-drupole time-of-flight mass spectrometry
GO	Gene Ontology	ADME	adsorption, distribution, metabolism and excretion
